# ORP/Osh mediate cross-talk between ER-plasma membrane contact site components and plasma membrane SNAREs

**DOI:** 10.1007/s00018-020-03604-w

**Published:** 2020-07-30

**Authors:** Marion Weber-Boyvat, Thorsten Trimbuch, Saundarya Shah, Jussi Jäntti, Vesa M. Olkkonen, Christian Rosenmund

**Affiliations:** 1 Institute of Neurophysiology and NeuroCure Cluster of Excellence, Charité Universitätsmedizin Berlin, 10117 Berlin, Germany; 2 Minerva Foundation Institute for Medical Research, 00290 Helsinki, Finland; 3 VTT Technical Research Center of Finland, 02044 VTT Espoo, Finland; 4 Department of Anatomy, Faculty of Medicine, University of Helsinki, 00014 Helsinki, Finland

**Keywords:** Osh, Sec9, ORP, SNAP-25, SNARE, Membrane contact site

## Abstract

OSBP-homologous proteins (ORPs, Oshp) are lipid binding/transfer proteins. Several ORP/Oshp localize to membrane contacts between the endoplasmic reticulum (ER) and the plasma membrane, where they mediate lipid transfer or regulate lipid-modifying enzymes. A common way in which they target contacts is by binding to the ER proteins, VAP/Scs2p, while the second membrane is targeted by other interactions with lipids or proteins.

We have studied the cross-talk of secretory SNARE proteins and their regulators with ORP/Oshp and VAPA/Scs2p at ER-plasma membrane contact sites in yeast and murine primary neurons. We show that Oshp-Scs2p interactions depend on intact secretory SNARE proteins, especially Sec9p. SNAP-25/Sec9p directly interact with ORP/Osh proteins and their disruption destabilized the ORP/Osh proteins, associated with dysfunction of VAPA/Scs2p. Deleting *OSH1-3* in yeast or knocking down ORP2 in primary neurons reduced the oligomerization of VAPA/Scs2p and affected their multiple interactions with SNAREs. These observations reveal a novel cross-talk between the machineries of ER-plasma membrane contact sites and those driving exocytosis.

**Electronic supplementary material:**

The online version of this article (10.1007/s00018-020-03604-w) contains supplementary material, which is available to authorized users.

## Introduction

Mammalian Oxysterol-binding proteins/OSBP-related proteins (ORPs) and yeast OSBP-homolog (Osh) proteins are cytosolic proteins characterized by a ligand-binding domain (ORD) with the ability to bind and/or transfer several oxysterols, cholesterol/ergosterol, phosphatidylinositol-4-phosphate [PI(4)P] or other phosphoinositides and phosphatidylserine [1–15]. Eight of the mammalian ORPs and three of the yeast Osh proteins contain a two phenylalanines in an acidic tract (FFAT) motif that mediates the interaction with the type II ER transmembrane protein, VAP (Scs2p in yeast), and thereby enables ER targeting [16, 17]. In addition, many of the ORP and Osh proteins have an N-terminal pleckstrin homology (PH) domain that binds to phosphoinositides and targets distinct non-ER organelle membranes [18–21]. This dual membrane targeting makes ORP/Osh proteins likely candidates to function at membrane contact sites (MCSs) between the ER and non-ER organelles [13, 22–26]. Targeting of ‘short’ OSBP homologues, like ORP2 and yeast Osh4p, lacking a PH domain to MCSs is less well understood, but is suggested to be mediated by patches of charged amino acids on the surface of the ORD [13].

**Fig. 1:**
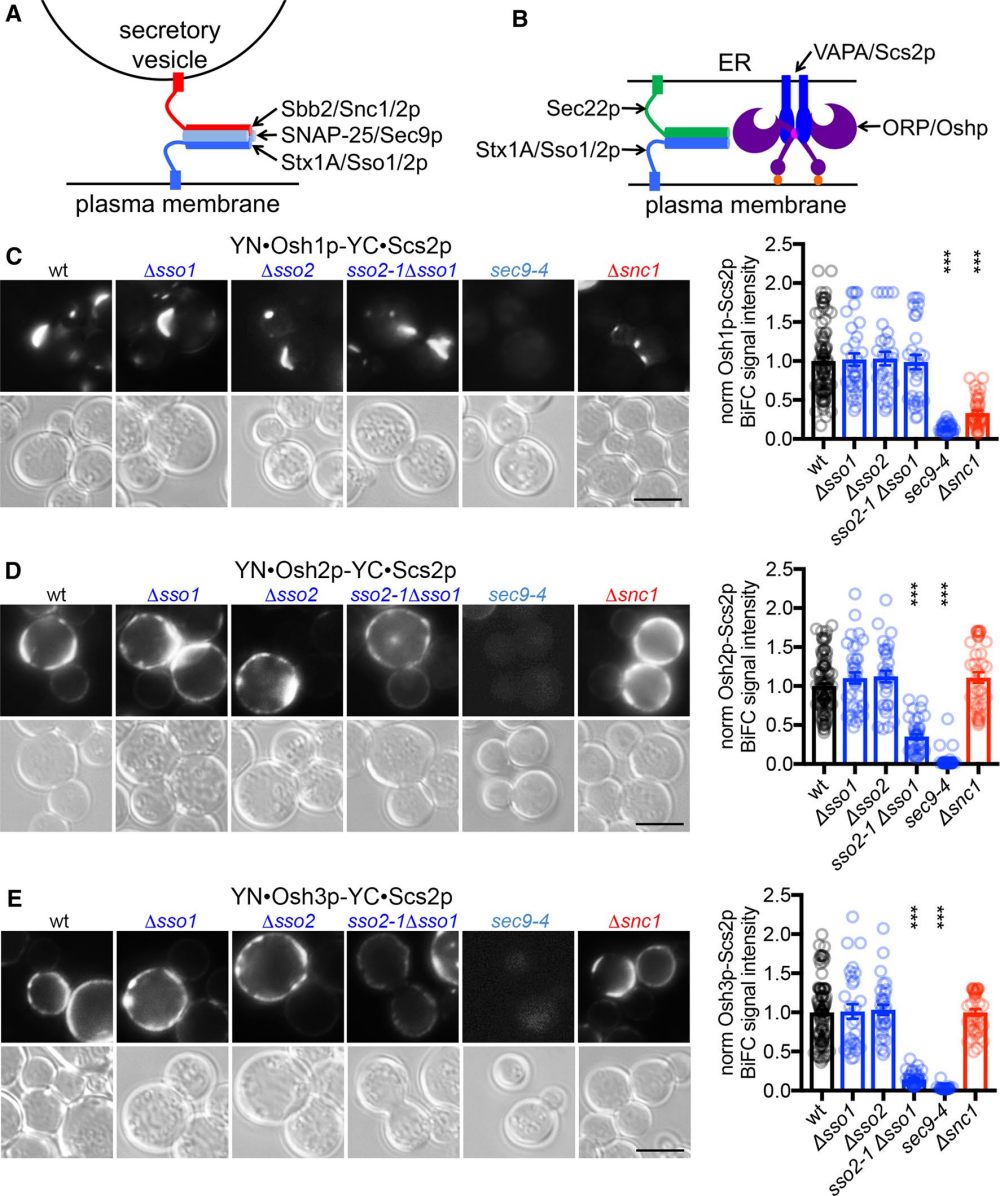
Yeast mutant strains for exocytic SNARE complex components show reduced Oshp-Scs2p BiFC interaction. **a** Schematic model of the exocytic mammalian/yeast SNARE complex consisting of the PM SNAREs SNAP-25/Sec9p (light blue) and Syntaxin1A/Sso1/2p (blue), and the vesicular SNARE Synaptobrevin2/Snc1/2p (red). **b** Schematic model of the mammalian/yeast ER-plasma membrane contact sites consisting of the PM SNARE Syntaxin1A/Sso1/2p (blue), the ER SNARE Sec22p (green) as well as the VAPA/Scs2p and ORP/Osh proteins (dark blue and purple). The FFAT motif in Osh/ORP proteins, mediating the interaction with VAPA/Scs2p, is highlighted in pink. Osh/ORP proteins target the plasma membrane due to their PH domain’s affinity for phosphoinositides (orange). **c**–**e** Live cell imaging of indicated vegetatively grown haploid yeast cells (wt, Y1; *Δsso1*, Y8, *Δsso2*, Y9; *sso2-1 Δsso1*, Y7; *sec9-4*, Y6; *Δscn1*, Y12) expressing YFP(N)·Osh1p (1362, C), YFP(N)·Osh2p (1363, D) or YFP(N)·Osh3p (1364, E) in combination with YFP(C)·Scs2p (1360). BiFC interactions were monitored by fluorescence microscopy in a minimum of 27 cells grown at 24 °C. The data represent mean ± SEM, ***p < 0.001. Scale bar, 5 µm

SNARE proteins constitute the minimal machinery for membrane fusion [27–29]. They are characterized by the SNARE motif, an α-helical region containing heptad repeats with a key residue that is either an arginine (R) or a glutamine (Q). Four SNARE motifs (3Q and 1R), typically present in one vesicle SNARE and two target compartment SNAREs assemble in trans to form a dense four-helix bundle, the SNARE complex, which brings the membranes to such close proximity that fusion can occur (Fig. 1a), [30, 31]. In vivo SNAREs induce exocytosis together with the exocyst complex, Rab family small GTPases and the Sec1/Munc18 (SM) proteins [32–37]. The zipping up of the SNARE complex, required for membrane fusion, has been proposed to be mediated by the SM proteins [34, 38]. A report by Petkovic et al. [39] proposed that the plasma membrane (PM) Q-SNARE Syntaxin1/Sso1p additionally acts with the ER R-SNARE protein Sec22p at ER-PM contacts, possibly in concert with ORP/Osh proteins, to execute a novel tethering function that does not involve fusion of the bilayers (Fig. 1b).

Yeast *Saccharomyces cerevisiae* has an extensive cortical ER that makes close contacts with the PM. The cortical ER presumably forms a barrier for exocytosis and endocytosis. Although endocytosis is spatially separated from cortical ER [40], sites of endo- and exocytosis are juxtaposed to the cortical ER [41, 42]. Therefore, membrane traffic and ER-PM contact sites may be coordinately regulated. In addition to suggested lipid transfer functions, Osh proteins have been linked to polarized growth in yeast due to their genetic and physical interactions with small GTPases and exocyst subunits [43, 44]. Osh4p, a ‘short’ Osh localizing to secretory vesicles, has been shown to reduce PI(4)P levels on the vesicles, suggesting that at least one Oshp influences exocytosis by altering the vesicle lipid composition [44, 45]. However, the role of the other Osh proteins in polarized growth has remained enigmatic, and the mechanisms coordinating exocytosis with ER–PM contacts, at which several Osh proteins are located, are thus far unknown [13].

In the present study we investigated the impact of proteins involved in exocytosis on the interaction of FFAT motif-containing Osh1-3p with Scs2p. We identified the Q-SNARE Sec9p as a novel Oshp interaction partner, and showed that this interaction is conserved in mammalian cells between ORP2 and SNAP-25, the Sec9p orthologue. Furthermore, we show that the mammalian ORP2 and yeast Osh1-3p mediate the oligomerization of VAPA/Scs2p and affect their multiple SNARE interactions. Our data suggest that the interplay of ORP/Osh proteins with SNAP-25/Sec9p and VAPA/Scs2p coordinates ER-PM contact sites.

## Materials and methods

### Yeast strains

The *Saccharomyces cerevisiae* yeast strains used are shown in Supplementary Table S1. When not otherwise stated, yeast cells were grown as described previously [46]. Y39 was generated by deletion of endogenous *PEP4* in the *sec9-4* mutant strain (Y6) using a hygromycin selection PCR cassette (1573).

### Plasmids

Plasmids used are listed in Supplementary Table S2. Yeast plasmids were generated as followed: A construct for expression of GFP-tagged *SCS2* (1577) was generated by PCR amplification of *SCS2* with added *Sma*I and *Sal*I sited followed by ligation into 1574. Yeast BiFC constructs were generated as followed. For constructing 1365, 1366, 1367 and 1368, *OSH4*, *OSH5*, *OSH6* and *OSH7*, respectively, were PCR amplified with added *Xma*I and *Xho*I sites and cloned into 1356. 1572 was generated by PCR amplifying *sec9-4* from genomic DNA (Y6) with added *Bam*HI and *Xho*I sites followed by ligation into 1358. To generate 1515 and 1516, *SEC22* was PCR amplified adding *Bam*HI and *Xho*I sites and cloning into 1356 and 1358, respectively. *SCS2* was PCR amplified with added *Bam*HI and *Sal*I sites and cloned into 1356, generating 1517. Additionally, *YC·SCS2* and *YC·SEC22* were cut out from 1360 and 1516, respectively, with *Spe*I and *Xho*I and cloned into 1565, generating 1566 and 1575. For overexpression studies with *OSH3*, *OSH3* was cut out from 1564 with *Spe*I and *Xho*I and cloned into 1565, generating 1567. To add HA tags to the YFP(N) BiFC constructs, a double HA was PCR amplified with added *Bam*HI/*Sma*I sites followed by ligation into the empty N-YN425ADH vector (1356), generating 1468. Subsequently, *OSH1*, *OSH2* and *OSH3* were cloned into this vector after releasing them from 1362, 1363 and 1364 with *Xma*I and *Xho*I, generating 1513, 1476 and 1477. A Flag tag was added to *YC·SCS2* (1360) by PCR amplification of a triple Flag tag with added *Bam*HI and *Eco*RI sites, followed by ligation into 1360, generating 1478. For bacterial production of His·Osh3p, it was PCR amplified with added *Spe*I and *Xho*I sites and cloned into 1509. For production of GST·Sec9p(421–651), *SEC9(421–651)* was cut out from 1530 with *Eco*RI/*Xho*I and cloned into 977.

Plasmids for experiments in mammalian cell lines were constructed as follows: For generation of mCherry tagged ORP constructs, hORP1L, hORP2 and hORP4L were released from 1226, 1227 and 1229 with *Bgl*II/*Kpn*I, and ligated into 1140, generating 1269, 1271 and 1276. For mCherry·OSBP (1274) creation, hOSBP was cut out from 1245 with *Bam*HI/*Hind*III and cloned into 1140. mCherry·ORP3 (1275) was constructed by releasing hORP3 from 1228 with *Hind*III/*Kpn*I and ligation into 1140. mCherry·ORP9 (1277) generation was achieved by cutting out hORP9 from 1234 with *Sac*I/*Sma*I and cloning into 1140. For hVAPA BiFC assays, hVAPA was cut out from 1223 with *Bgl*II/*Kpn*I and ligated into 1196, generating 1560.

### Virus constructs and production

The lentiviral plasmid pFUGW [47] served as standard backbone in all used lentiviral shuttle vectors. The following modifications were made: first, to obtain neuron restricted expression, the human ubiquitin promoter in pFUGW was replaced by the human synapsin-1 promoter and second, to observe viral infection efficiency, the expression cassettes was constructed as polycistronic construct by included a nuclear localized fluorescence reporter (GFP or RFP) coupled via a self-cleaving P2A site [48] to the gene of interest.

For neuronal expression of human ORP1L and ORP2 the corresponding cDNAs (NCBI accession number NM080597.4 and NM144498.3, respectively) were cloned into the modified pFUGW to form BL1209 f(syn)NLS.GFP-P2A-hORP1L-w and BL1210 f(syn)NLS.GFP-P2A-hORP2-w. To reduce lentivirally the endogenous expression level of mammalian ORP2, a transcription cassette was inserted upstream of a human synapsin 1 promoter controlled NLS.RFP reporter that contains the human Polymerase III U6 promoter which drive the transcription of a shRNA hairpin against the OPR2 sequence 5′-GCAGCTATGGATAGAGCAG-3′ using the 5′-TACTCGAGTA-3′ linker sequence (BL1332 f(U6)shRNA-ORP2-(Syn)NLS.RFP-w). A scrambled shRNA against rat clatrin heavy chain, served as negative control (BL360 f(U6)shRNA-scrCHC-(Syn)NLS.RFP-w, Kononecko et al. 2014). To visualize oligomerization of VAPA by BiFC the human VAPA transcript (NM194434.3) was N-terminally tagged by Venus(N) and Venus(C) parts via a 3xGGGS linker and cloned into the lentiviral vector forming BL1310 f(syn)NLS.RFP-P2A-VN.hVAP.A-w and BL1356 f(syn)NLS.RFP-P2A-VC.hVAP.A-w, respectively. For the SNAP-25 expression in SNAP-25 KO cells, the SNAP-25 cDNA (GenBank: M22012.1) was cloned into the modified pFUGW to generate BL-940 (f(syn)NLS-RFP-P2A-mSNAP25w). Cre mediated deletion of Syntaxin1A/B was accomplished with the published vector BL-150 f(syn)iCreRFP-P2A-w [49]. The non-cre expressing, empty vector served as control (BL-181) f(syn)NLS-RFP-P2A). To visualize exogenous FLAG-tagged VAPA by western blotting using the VAPA antibody (Fig. 6), VAPA was N-terminally tagged by a 64 aa peptide of the VN BiFC (aa105-173) leading to a size shift of 10 kDa than compared to endogenous VAPA. All final constructs were verified by both restriction digest and sequencing.

All lentiviral particles were provided by the Viral Core Facility of the Charité -Universitätsmedizin Berlin (vcf.charite.de) and were prepared as described previously Lois et al. [47]. Briefly, HEK293T (RRID: CVCL_0063) were co-transfected with the shuttle vector (10 mg) and helper plasmids, pCMVdR8.9 and pVSV.G (5 mg each) with polyethylenimine.cells. Virus containing cell culture supernatant was collected after 72 h and filtered for purification. Aliquots were flash-frozen in liquid nitrogen and stored at − 80 °C. Neurons were infected with lentivirus at DIV 3 or DIV 7.

### Culture of mammalian HEK293 cells and primary hippocampal neurons

HEK293 cells were cultured in DMEM (Dulbecco’s Modified Eagle Medium; Sigma-Aldrich) supplemented with 10% fetal bovine serum (FBS), 100 U/ml penicillin, and 100 μg/ml streptomycin.

All procedures concerning mice use and maintenance were approved by the Animal Welfare Committee of Charité Medical University and the Berlin State Government. For all experiments, primary hippocampal neurons from P0-P2 newborn mice were cultured on 1–3-week-old astrocyte feeding layers (derived from P0-P2 mice cortices and plated on collagen/poly-D-lysine microislands on agarose-coated coverslips for autaptic cultures or on collagen/poly-D-lysine coated wells for continental cultures). In case of the SNAP-25 KO mice hippocampal neurons were prepared form E18 mice. For continental cultures, glia proliferation was arrested by the addition of antimitotic agent 8 µM 5-fluoro-2-deoxyuridine and 20 µM uridine (FUDR) to the astrocyte media. Neurons were plated at a density of 3000 neurons per 35 mm well for autaptic cultures and 100,000 neurons per 35 mm well for continental cultures and grown in Neurobasal-A media containing B-27 supplement and Glutamax (Invitrogen, Germany). In all experiments, neurons were incubated at 37 °C for 7 or 14 days prior further analysis.

### Fluorescence microscopy of yeast cells

Yeast cells were grown to OD_600_ 0.8–1 at the permissive temperature (24 °C) in SC-ura-leu or SC-ura-leu-his medium. Cells were observed using a Zeiss Axio Observer Z1 microscope with an ECPlnN 53x/1.4 Oil objective and Colibri laser. Images were recorded with the Axio Vision Rel. 4.8.1 software (Carl Zeiss Imaging Solutions GmbH). The exposure time for the BiFC of YFP was 2–5 s for Oshp-Sec9p interaction and 1–2 s for Oshp-Scs2p, Scs2p-Scs2p, Scs2p-Sec9p, Scs2p-Sec22p, Scs2p-Sso1p, Sec22p-Sso1p, Mso1p-Sec1p interaction. Image panels were prepared using Adobe Photoshop7 software. Quantification of the signal intensities were performed with the original images obtained with the same microscope and the same exposure time. For each condition the signals in at least 20 cells were quantified by measuring the mean gray value of random points on the plasma membrane or same size boxes compromising the whole cell using ImageJ 1.42. The background fluorescence of 10 random points in each condition was subtracted from the obtained measurements.

### Fluorescence microscopy of HEK293 cells

HEK293 cells were grown to about 70% confluence and transfected with the BiFC plasmids and the co-transfection marker using Lipofectamine™ 2000 (Invitrogen). After 24-h incubation, cells were fixed with 4% paraformaldehyde in phosphate buffered saline, and visualized with a Zeiss Axio Observer Z1 microscope with an ECPlnN 40x/0.75 DICII objective and Colibri laser and recorded with the Axio Vision Rel. 4.8.1 software. The exposure time for the BiFC of Venus was 1–2 s and of the co-transfection markers mCherry or mCherry-ORP 0.5–1 s. Image panels were prepared using Adobe Photoshop7 software.

### Immunofluorescence staining of neurons and HEK293 cells

After cell fixation at DIV 14 (4% paraformaldehyde in PBS, 7 min), cells were permeabilized (0.1% Triton X-100/PBS, 3 × 15 min, PBST) and unspecific binding was blocked (5% normal goat serum (NGS) /PBST, 1 h). Primary antibody incubation was performed in 5% NGS/PBST overnight at 4 °C. After washing with PBST (3 × 5 min), secondary antibody incubation was performed with Alexa Fluor-conjugated antibodies in PBST, 1 h at room temperature. The cells were washed 2 × 10 min with PBST and 1 × 10 min PBS and mounted in Mowiol (SIGMA-ALDRICH). Cells were visualized with a Olympus IX81 epifluorescent microscope, MicroMax:1300YHS camera (Princeton Instruments) and MetaMorph software (Molecular Devices). The exposure time for the BiFC of Venus was 0.5–1 s. Image panels were analyzed with the Fiji Software and prepared using Adobe Photoshop software.

### Immunoprecipitation of HA·Osh1p and HA·Osh3p

Immunoprecipitations were performed essentially as described previously [50]. An isogenic strain lacking the tagged protein served as negative control. For Western blot analysis proteins were transferred onto nitrocellulose membranes and bound antibodies were visualized with the ECL detection system (Pierce). For quantifications immunoprecipitations were performed for at least three times, analyzed using Bio-Rad Quantity One software and normalized for the amount of immunoprecipitated HA·Osh3p.

### Immunoprecipitation of FLAG·VAPA

At DIV 7 or 14, cells were washed and resuspended in 200 µl ml lysis buffer per well (50 mM Tris HCl pH 7.9, 150 mM NaCl, 1% NP-40, Protease inhibitor cocktail, Roche Diagnostics). Cell breakage was achieved by shearing in lysis buffer, 30 min incubation at 4 °C, and followed by 10 min centrifugation at 13,000 rpm in a microcentrifuge to remove unbroken cells. The obtained lysates were incubated with 15 µl anti-FLAG® M2 magnetic beads (SIGMA-ALDRICH) for 20 min at room temperature. The beads were then washed three times with lysis buffer, resuspended in 30 µl l × LSB and boiled for 5 min. For Western blot analysis, proteins were transferred onto nitrocellulose membranes. Bound antibodies were visualized with the ECL detection system (Thermo Scientific).

### Hsp150p secretion

Yeast strains were grown at 24 °C to A_600_ 0.3–0.5. Cells were collected, washed, adjusted to A_600_ 0.3 in fresh medium and divided to 24 °C, 34 °C or 37 °C. Samples were removed after 1 h and 2 h incubation and NaN_3_ was immediately added to a final concentration of 10 mM to the samples cooled on ice. Cells were pelleted at 4 °C and 30 µl of the supernatants were analyzed by western blotting with anti-Hsp150p antibodies.

### Protein production

GST·SNAP-25, GST·Sec9p(421–651) and GST·VAPA(ΔTM) were expressed in *E.coli* and purified on Gluthatione-Agarose 4B (Macherey-Nagel). The resin-bound proteins were stored in PBS at 4 °C as a 50% slurry. GST was produced and purified in the same way. His-tagged Osh3p, pFOLD·ORP2 (J. Peränen, University of Helsinki, Finland) and VAPA(ΔTM) were purified on Ni-NTA agarose (Invitrogen). All His-tagged proteins were concentrated in Amicon Ultra (Millipore) in PBS.

### In vitro* binding assays*

For in vitro pull-down experiments between ORP2 and SNAP-25, 1 μM GST·SNAP-25 or GST were combined with equimolar amounts of pFOLD·ORP2 and 20 µl of Gluthation-Agarose. The mixtures were incubated at room temperature in a total volume of 100 μl PBS for 1 h with gentle mixing. After the incubation, the resin was washed three times with 1 ml PBS. The bound proteins were released into 60 μl of 1 × LSB by heating for 5 min at 95 °C and subjected to SDS-PAGE and Coomassie Blue staining.

In in vitro pull-down experiments between Osh1/3p and Sec9p(412–651), 2 μM GST·Sec9p(421–651) or GST immobilized on Glutathione-Agarose was used. Equal molar amounts of His·Osh3p were added to the proteins in a total of 100 µl PBS + 0.1% TritonX-100. After 1 h of incubation with gentle mixing, the resin was washed three times with 1 ml PBS and the bound proteins were released by heating for 5 min at 95 °C into 60 μl of 1 × LSB and subjected to SDS-PAGE and Coomassie Blue staining.

### In vitro* VAPA oligomerization assays*

For in vitro VAPA oligomerization experiments, 1 μM GST·VAPA(ΔTM) immobilized on Glutathione-Agarose was incubated with the same amount of His-VAPA(ΔTM) (0.4 µM) and increasing amount of His·ORP2 (0, 0.2 µM) for 1 h in a total of 200 µl PBS and 0.05% Triton X-100. After the incubation, the resin was washed (3 × 1 ml PBS) and the released proteins (40 μl of 1 × LSB, 5 min at 95 °C) were subjected to SDS-PAGE and Coomassie Blue staining.

### Antibodies

The HA-tag antibodies were purchased from Pierce, the FLAG-tag antibodies from SIGMA-ALDRICH and the anti-GFP antibodies from Thermo Scientific/Molecular Probes. The anti-SNAP-25, Stx1A, Stx1B, MAP2 and vGlut1 antibodies were purchased from Synaptic Systems, the β-Tubulin antibodies from SIGMA-ALDRICH and the VAPA antibodies from abcam. Antibodies against ORP1L and ORP2 have been characterized previously [20, 51]. The anti Sec9p antibody was obtained from Dr. Patrick Brennwald (University of North Carolina at Chapel Hill, USA). The anti-Sso1/2p (K8) antibody [52] has been published previously. The anti-Hsp150p antibody [53] was a kind gift from Prof. Marja Makarow (University of Helsinki). The anti-Snc1p and Snc2p antibodies were obtained from Prof. Michael Knop (ZMBH of the University of Heidelberg, Heidelberg, Germany).

Alexa Fluor conjugated secondary antibodies for immunofluorescence staining were purchased from Jackson ImmunoResearch.

### Statistical testing

All statistical analyses were done using Prism (Graphpad). For details see Supplementary Table S3.

## Results

### Yeast mutant strains for exocytic SNAREs show reduced Oshp-Scs2p BiFC interaction

To identify potential links between the machineries involved in vesicle fusion (Fig. 1a) and ER-PM contacts (Fig. 1b); [39], we studied the proximity of the FFAT motif-containing Osh1p, Osh2p and Osh3p with their ER receptor Scs2p in different temperature-sensitive yeast mutants functionally linked to exocytosis [52, 54]. As readout we applied the method of Bimolecular Fluorescence Complementation (BiFC) [55, 56] that uses N- and C-terminal parts of YFP tagged to Osh1-3p and Scs2p. High BiFC signal relates to close proximity. The BiFC signal between all three of Osh1-3p and Scs2p, which in wild type cells localized at the nucleus-vacuole junction (NVJ) (Osh1p) [23, 57] or cortical ER (Osh2/3p) [26], was almost completely lost in cells carrying a mutation in Sec9p (G458D), a Q-SNARE with a key role in exocytosis in yeast and an orthologue to mammalian SNAP-25 (Fig. 1c–e). The Sec9p(G458D) mutation has been shown to result in less binary and ternary SNARE interactions in vitro, decreased secretion and increased temperature sensitivity [58, 59]. Noteworthy, while previous studies only identified Syntaxin-1 and Sec22 homologues to be involved in ER-PM contacts (Fig. 1b); [39], this finding indicates a contribution of the SNAP-25 orthologue Sec9p to ER-PM contacts. Furthermore, signals between Osh2p/Osh3p and Scs2p were partially dependent on the Syntaxin-1A orthologue Sso1/2p (Fig. 1d, e), while the signal between Osh1p and Scs2p, which targets the Golgi and nucleus-vacuole contact sites, partly depended on the Synaptobrevin orthologue Snc1p (Fig. 1c). In accordance, co-immunoprecipitations with HA-Oshp revealed that while Osh3p associates with Sso1/2p, Osh1p co-immunoprecipitates Snc1/2p, suggesting different protein complexes (Supplementary Figure S2A and C). At the same time, the BiFC interaction signal between Osh1-3p and Scs2p did not depend on accessory proteins that mediate SNARE complex assembly or disassembly, Sec4p, Sec18p, Mso1p, Sec1p or Sro7p (Supplementary Figure S1), thus suggesting a distinct novel function of the SNARE proteins, especially the SNAP-25 orthologue Sec9p, in concert with the Osh proteins.

### Osh proteins are selectively degraded in the sec9-4 SNARE mutant strain and interact with the SNARE motif region of Sec9p

**Fig. 2:**
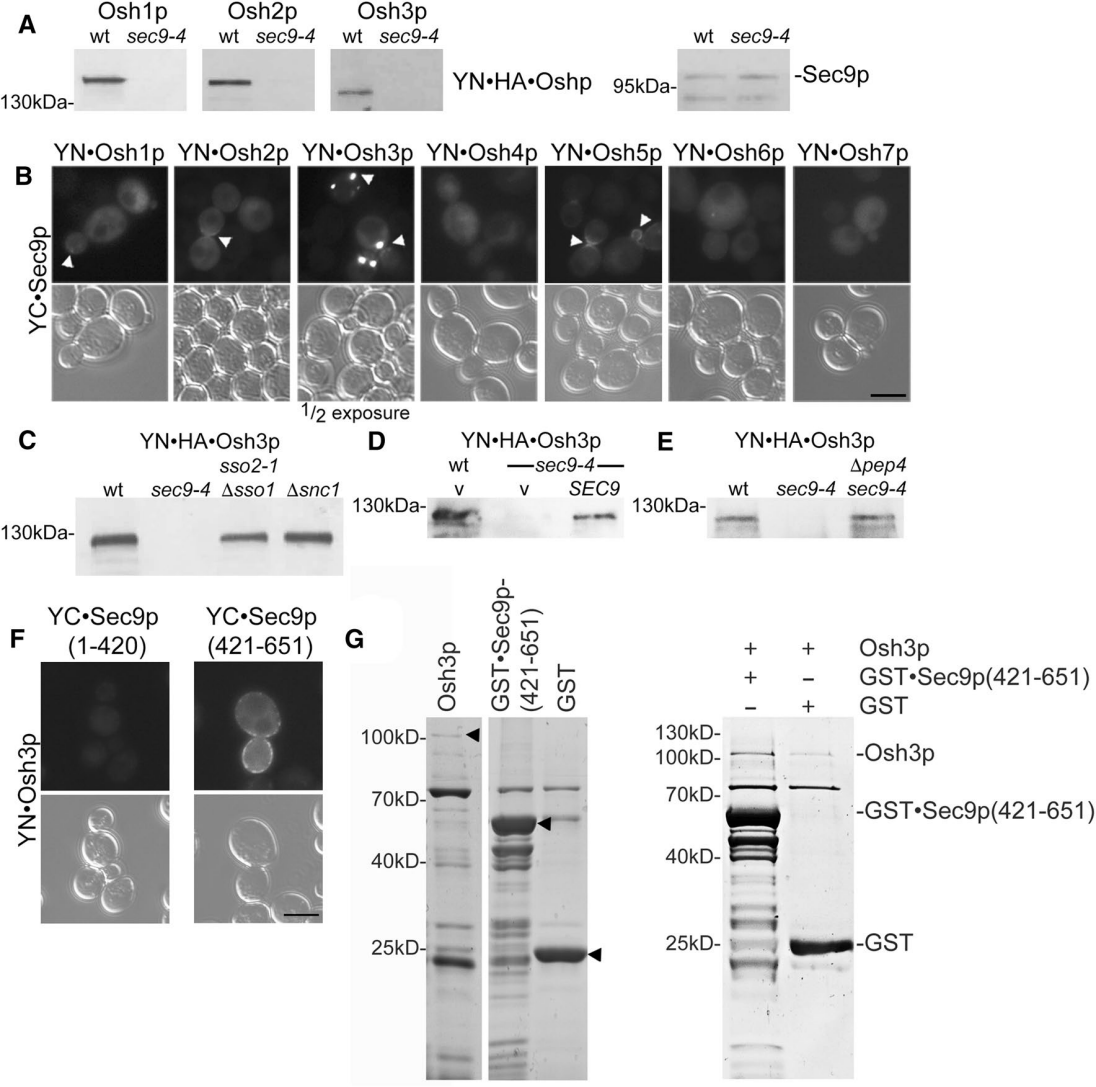
Osh proteins are selectively degraded in the *sec9-4* SNARE mutant strain and interact with the SNARE motif region of Sec9p. **a** FFAT motif containing Osh proteins are diminished in the *sec9-4* yeast mutant stain. SDS lysates of wt (Y1) or *sec9-4* yeast mutant (Y6) cells transformed with the indicated *OSH* constructs (*OSH1*, 1362; *OSH2*, 1363; *OSH3*, 1364). As a control Sec9-4p protein level were examined. SDS lysates were analyzed by Western blotting and detection was performed with anti-HA and -Sec9p antibodies. **b** The Oshp-Sec9p BiFC interaction in vivo*.* Haploid yeast cells (Y1) were transformed with the indicated constructs (YFP(N)·Osh1p, 1362; YFP(N)·Osh2p, 1363; YFP(N)·Osh3p, 1364; YFP(N)·Osh4p, 1365; YFP(N)·Osh5p, 1366; YFP(N)·Osh6p, 1367; YFP(N)·Osh7p, 1368; YFP(C)·Sec9p, 1404) and vegetatively grown at 24 °C prior to examination for BiFC interaction by fluorescence microscopy. Arrowheads point to Oshp-Sec9p BiFC interactions at sites of secretion (emerging or growing bud and septum). In case of the Osh3p-Sec9p interaction the exposure time of the image is half. Scale bar, 5 µm. **c** Osh3p is not degraded in other SNARE complex deficient mutant yeast strains. SDS lysates of yeast cells (wt, Y1; *sso2-1 Δsso1*, Y7; *sec9-4*, Y6; *Δscn1*, Y12) expressing *OSH3* (1364). The detection was performed as in (**a**). **d** The degradation effect of Osh3p is rescued by reintroduction of wt *SEC9*. SDS lysates of *sec9-4* yeast mutant (Y6) cells transformed with an empty vector or wild type *SEC9* and *OSH3* (vector, 1358; *SEC9*, 1405; *OSH3*, 1477). The detection was performed as in (**a**). **e** Osh3p is degraded via the vacuolar pathway. SDS lysates of wt (Y1), *sec9-4* (Y6) or *sec9-4 Δpep4* (Y39) yeast cells transformed with an *OSH3* (1477) construct. The detection was performed as in (**a**). **f** Osh3p shows BiFC interaction with the Sec9p SNARE motifs (amino acid 421–651) and not with the Sec9p N-terminal domain (amino acid 1–420) in vivo. Live cell imaging of vegetatively grown haploid yeast cells (Y1) transformed with YFP(N)·Osh3p (1364) in combination with YFP(C)·Sec9p(1–420) (1527) or YFP(C)·Sec9p(421–651) (1525). Scale bar, 5 µm. **g** Osh3p directly interacts with Sec9p(421–651) in vitro. GST·Sec9p(421–651) or GST (2 μM) bound on Glutathione-agarose were mixed with 0.1% Triton X-100 and equal molar amounts of Osh3p, and incubated at room temperature for 1 h. Bound proteins were separated by SDS–PAGE and stained with Coomassie blue. Purified proteins (arrowheads) are shown on the left panel

When searching for an explanation for the loss of the BiFC signal between Osh1-3p and its ER receptor Scs2p, we observed that the applied tagged Osh proteins were selectively lost, possibly degraded, in the *sec9-4* SNARE mutant strain (Fig. 2a). Intriguingly, in wild type cells we observed close proximity of Osh2p, Osh3p and Osh5p (the only other Oshp which is also degraded in the *sec9-4* strain, data not shown) and to a lesser extent Osh1p with Sec9p at the sites of secretion, the growing bud and the septum (Fig. 2b, arrowheads). Compared to the other Osh proteins, Osh3p showed the strongest BiFC interaction signal with Sec9p at large puncta likely representing previously reported secretory vesicle clusters (Fig. 2a); [60]. We therefore continued to focus predominantly on Osh3p.

The observed loss of the Osh3 protein in the *sec9-4* strain was specific for Sec9p, as Osh3p levels were stable in other SNARE protein mutant strains (Fig. 2c) and could be partially restored by reintroduction of low expression wt *SEC9* in the *sec9-4* mutant strain (Fig. 2d). It has previously been suggested that Osh1p is primarily degraded in the vacuolar lumen [61]. We therefore examined the role of vacuolar degradation in the turnover of Osh3p in *sec9-4* mutant cells, and found that removing the key vacuolar aspartyl protease (*pep4*Δ) restored the Osh3 protein level (Fig. 2e), indicating that Osh3p was degraded in the vacuole of *sec9-4* mutant cells.

Sec9p is composed of an amino-terminal domain (amino acids 1–420) only present in the yeast protein and a well-conserved SNARE motif domain (amino acids 421–651) [59]. To better understand the mechanism and importance of the Oshp-Sec9p interaction we investigated which part of Sec9p mediates the interaction with Osh3p. Osh3p produced a BiFC signal with the Sec9p SNARE motif domain, while negligible BiFC signal, similar to non-transfected yeast cells, was detected with the N-terminus of Sec9p (Fig. 2f). The different localization of the Sec9p(421–651)-Osh3p BiFC interaction site compared to full length Sec9p (Fig. 2b) is likely due to missing targeting domains located in the Sec9p N-terminus. The interaction between Osh3p and the SNARE motif domain area of Sec9p was further confirmed by in vitro pull-down experiments with purified His_6_-tagged Osh3p and GST-tagged Sec9p(421–651), which revealed specific co-purification of Osh3p with GST·Sec9p(421–651) (Fig. 2g). Only minor background Osh3p signal was observed with GST alone. These results support the BiFC results and suggest a direct interaction between the Osh proteins and the SNARE motif domain of Sec9p. Noteworthy, even though Sec9p can directly interact with Osh3p, in vivo co-immunoprecipitation experiment using different SNARE complex modulating mutants suggested that the binary SNARE complex proteins Sec9p and Sso1/2p are in fact in one complex interacting with Osh3p in vivo (Supplementary Figure S2A and B).

### The Oshp-Sec9p interplay is conserved in mammalian cells

**Fig. 3:**
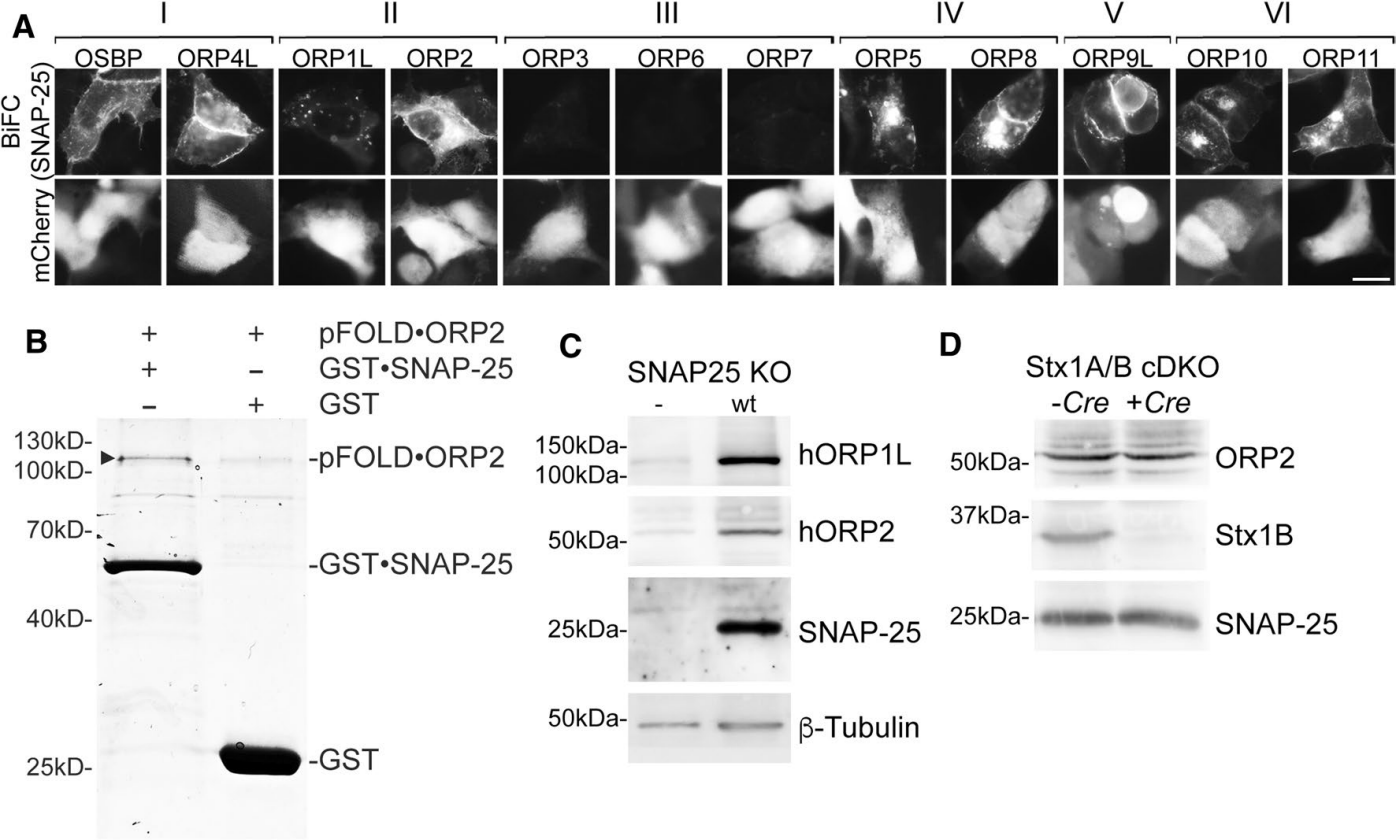
The Oshp-Sec9p interplay is conserved in mammalian cells. **a** The mammalian Oshp homologues of the families I, II, IV, V and VI show BiFC interaction with SNAP-25, the mammalian orthologue of Sec9p, at the plasma membrane in vivo*.* Fluorescence imaging of HEK293 cells transfected with plasmids expressing indicated Venus(N)·ORP constructs (OSBP, 1245; ORP4L, 1229; ORP1L, 1226; ORP2, 1227; ORP3, 1228; ORP6, 1231; ORP7, 1232; ORP5, 1230; ORP8, 1233; ORP9, 1234; ORP10, 1235; ORP11, 1236) in combination with Venus(C)·SNAP-25 (1239) and the co-transfection marker mCherry (1140). Note: All ORPs are partially shifted to the plasma membrane by BiFC interaction with SNAP-25, except ORP1L. Scale bar, 10 µm. **b** ORP2 interacts directly with SNAP-25 in vitro using purified proteins. GST·SNAP-25 or GST were mixed with pFOLD·ORP2 (all 1 μM) and Glutathione-agarose. After 1 h incubation at room temperature bound proteins were released, separated by SDS–PAGE and stained with Coomassie blue. **c** ORP1L and ORP2 are greatly diminished in SNAP-25 KO mice hippocampal neurons. SDS lysates of SNAP-25 KO mice with lentivirus-based reintroduction of an empty vector (BL181) or wt SNAP-25(BL940) and overexpression of hORP1L (BL1209) and hORP2 (BL1210). Cells were transduced at DIV 1 and lysed at DIV 16. SDS lysates were analyzed by Western blotting and detection was performed with anti-ORP1L, -ORP2, -SNAP-25 and -β-Tubulin antibodies. **d** Endogenous ORP2 protein levels are unaltered in Stx1B^FL/FL^/Stx1A KO mice. SDS lysates of Stx1A/B cDKO mice hippocampal neurons infected at DIV 1 with viruses to express *Cre* recombinase (BL150) or an empty vector (BL181). Cells were transduced at DIV 1 and lysed at DIV 16. SDS lysates were analyzed by Western blotting and detection with anti-ORP2, Stx1B and SNAP-25 antibodies

The evolutionary conservation of Oshp and Sec9p proteins (ORPs and SNAP-25 in humans) prompted us to check for proximity between the mammalian homologues. Nine of the 12 human ORPs (OSBP, ORP4L, ORP1L, ORP2, ORP5, ORP8, ORP9L, ORP10 and ORP11) produced a positive BiFC signal with SNAP-25 in HEK293 cells (Fig. 3a). The signals were localized predominantly to the plasma membrane (OSBP, ORP4L, ORP2) or to additional intracellular structures (ORP5, ORP8, ORP9L, ORP10, ORP11), except for ORP1L which targets late endosomes/lysosomes via the GTPase Rab7 [20]. ORP proteins of subfamily III (ORP3, − 6 and − 7) did not show any BiFC signal with SNAP-25 (Fig. 3a). Since most of the ORPs produced a BiFC signal with SNAP-25, suggesting that the interaction sites resides in a very conserved domain like the ORD, we studied the interaction between the ‘short’ ORP2 and SNAP-25 in vitro using purified proteins produced in *E.coli*. In this assay ORP2 bound to GST·SNAP-25 to a greater degree than to GST alone (Fig. 3b), supporting a direct interaction of the two proteins.

In yeast Osh proteins that showed BiFC interaction with Sec9p were degraded in the *sec9-4* strain. To test if the same holds true in mammalian cells we employed hippocampal neurons from SNAP-25 KO mice into which wt SNAP-25 was reintroduced. Lentivirally transduced human ORP2 and ORP1L were greatly diminished in SNAP-25 KO cells, while they persisted in wt SNAP-25 expressing cells (Fig. 3c). In Stx1A/B cDKO mouse hippocampal neurons, ORP2 protein levels were unaltered when Stx1B was removed by *Cre* recombinase (Fig. 3d), suggesting that also in mammalian cells the degradation of ORP2 occurs specifically in the absence of functional SNAP-25. These results suggest that the ORP/Osh proteins may function in association with SNAP-25/Sec9p in an evolutionary conserved manner.

### The yeast sec9-4 cells display disturbed Scs2p oligomerization and Sec22p-Sso1p interaction

**Fig. 4:**
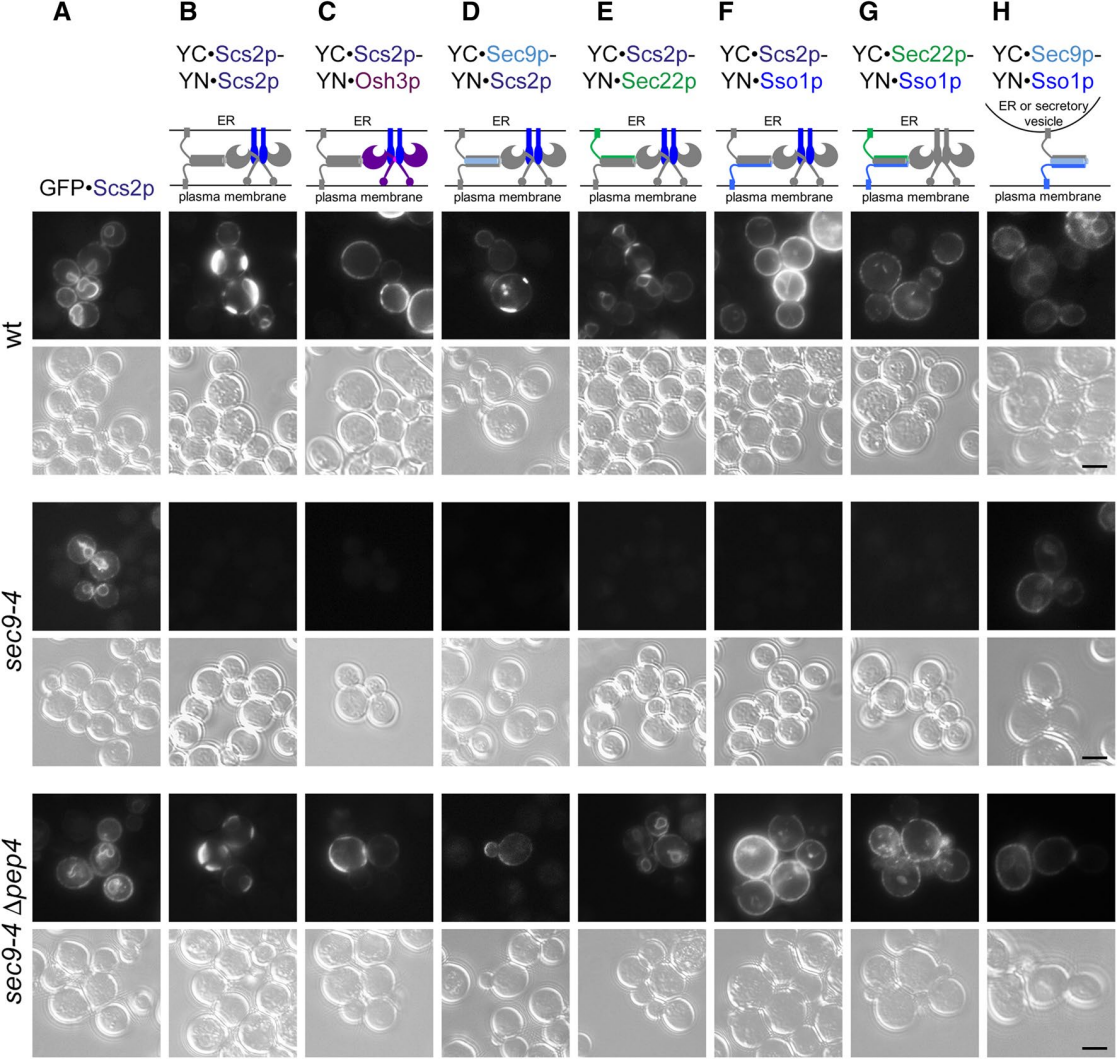
Scs2p BiFC interactions with itself, the ER SNARE Sec22p and the plasma membrane SNARE Sso1p are disturbed in the *sec9-4* SNARE mutant strain in vivo*.* Additionally, the Sec22p-Sso1p BiFC interaction is lost. Live cell imaging of vegetatively grown yeast cells at the permissive temperature (wt, Y1; *sec9-4*, Y6; *sec9-4 Δpep4*, Y39) transformed with the indicated constructs shown as a schematic above the respective panels. In detail: (**a**) GFP·Scs2p, 1577; (**b**) YFP(C)·Scs2p, 1360; YFP(N)·Scs2p, 1517; (**c**) YFP(C)·Scs2p, 1360; YFP(N)·Osh3p, 1364; (**d**) YFP(C)·Sec9p, 1404 or YFP(C)·Sec9-4p, 1572; YFP(N)·Scs2p, 1517; (**e**) YFP(C)·Scs2p, 1360; YFP(N)·Sec22p, 1515; (**f**) YFP(C)·Scs2p, 1360; YFP(N)·Sso1p, 1395; (**g**) YFP(C)·Sec22p, 1516; YFP(N)·Sso1p, 1395; (**h**) YFP(C)·Sec9p, 1404 or YFP(C)·Sec9-4p, 1572; YFP(N)·Sso1p, 1395. Note that in case of the *sec9-4* stain the YFP(C)·Sec9-4p construct was used. BiFC interaction was examined by fluorescence microscopy. Scale bar, 5 µm

The *sec9-4* SNARE mutant strain is characterized by the loss of Osh1-3p (Fig. 1). To understand the consequences of this mutation (G458D) [58] for the ER-PM contacts, we investigated the localization and interactions associated with Osh3p and the ER-PM SNARE complex proteins in this mutant strain using BiFC. A control, the GFP·Scs2p fusion protein, showed normal wt-like localization in the *sec9-4* strain (Fig. 4a top and middle row) even though the Osh3p-Scs2p signal was lost due to the degradation of Osh3p (Figs. 2a and 4c top and middle row). However, compared to the wt strain, in the *sec9-4* strain Scs2p failed to produce most of the BiFC signals. Not only did Scs2p fail to interact with the plasma membrane SNAREs Sec9p/Sec9-4p and Sso1p and the ER SNARE Sec22p (Fig. 4d–f top and middle row), but it also failed to produce a BiFC signal with itself (Fig. 4b top and middle row). The BiFC signal of Sso1p with Sec22p was also lost (Fig. 4g top and middle row), whereas the BiFC signal between Sso1p and Sec9p/Sec9-4p, possibly reflecting both SNAREs involved in ER-PM membrane contact and secretory SNARE complexes, was largely unaffected (Fig. 4h top and middle row).

Blocking vacuolar degradation by the deletion of *PEP4* in the *sec9-4* mutant strain restored the Osh3p protein level (Fig. 2e) and all BiFC signals to wt levels (Fig. 4, bottom row). This strongly suggests that the observed loss of BiFC signals in the *sec9-4* strain is predominantly caused by the loss of Osh proteins.

### FFAT motif-containing Osh proteins regulate Scs2p oligomerization and SNARE association

**Fig. 5:**
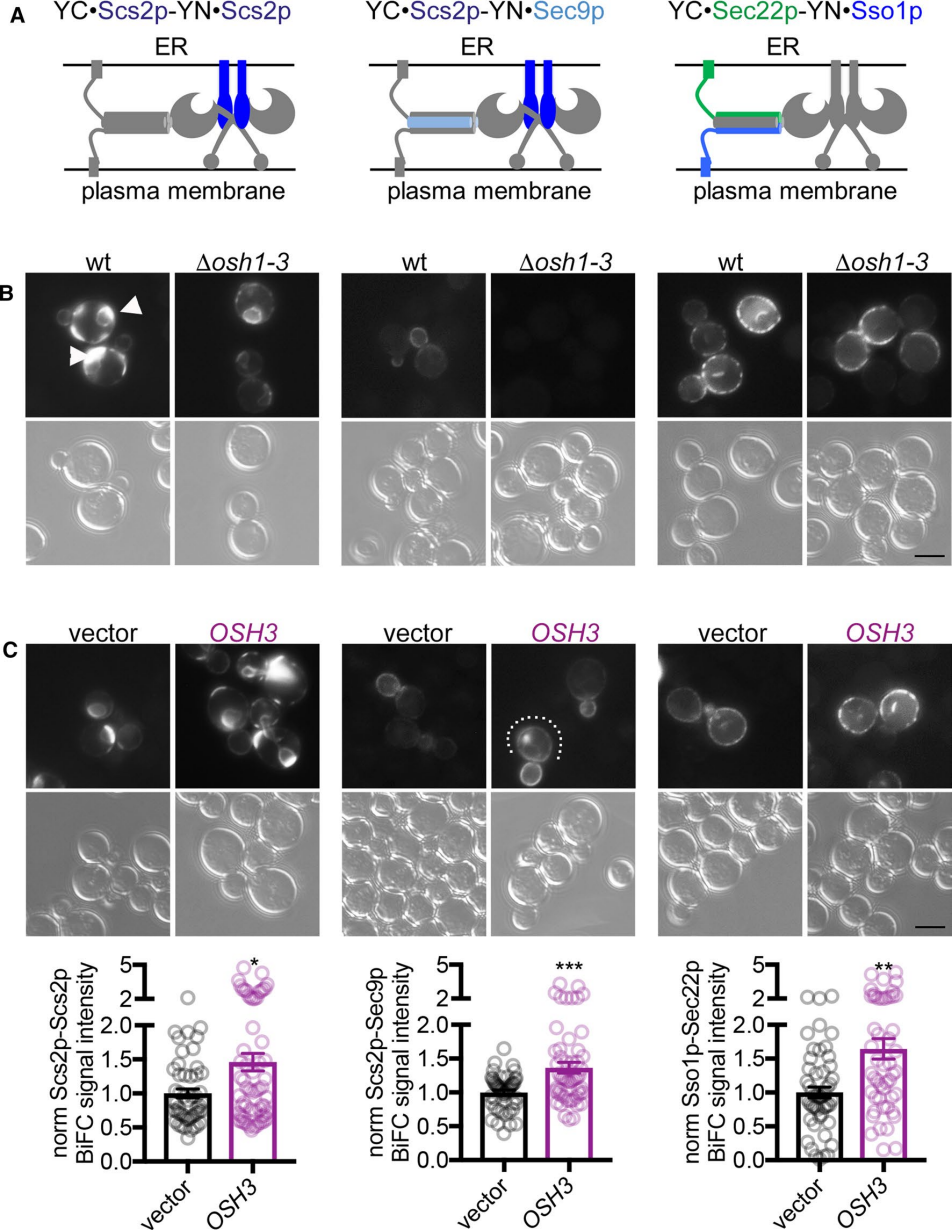
FFAT motif containing Osh proteins regulate Scs2p oligomerization and SNARE association. **a** Schematic representation of constructs used for the BiFC assays below. **b** Osh1-3p are important for Scs2p-Scs2p and Scs2p-Sec9p plasma membrane BiFC interaction. Live cell imaging of vegetatively grown wt (Y2) or *Δosh1-3* (Y36) yeast cells transformed with the indicated constructs (YFP(C)·Scs2p, 1566; YFP(N)·Scs2p, 1517; YFP(C)·Sec22p, 1575; YFP(N)·Sso1p, 1395; YFP(N)·Sec9p, 1399) and examined for BiFC interaction by fluorescence microscopy. The arrowheads point to Scs2p oligomers at plasma membrane patches in wt cells. Scale bar, 5 µm. **c** *OSH3* overexpression results in increased Scs2p-Scs2p, Scs2p-Sec9p and Sec22p-Sso1p BiFC interaction. Live cell imaging of vegetatively grown wt yeast cells (Y38) transformed with an empty vector (1565) or *OSH3* (1567) in combination with the indicated BiFC constructs (YFP(C)·Scs2p, 1360; YFP(N)·Scs2p, 1517; YFP(C)·Sec22p, 1516; YFP(N)·Sso1p, 1395; YFP(N)·Sec9p, 1399). Cells were examined for BiFC interaction by fluorescence microscopy. Note the partial re-localization of the Scs2p-Sec9p BiFC interaction site towards the mother cell upon *OSH3* overexpression (dotted line). BiFC signal intensities were analyzed in a minimum of 49 cells per interaction mode. The data represent mean ± SEM, *p < 0.05, **p > 0.01, ***p < 0.001. Scale bar, 5 µm

The degradation of Osh proteins and the resulting loss of BiFC signals within the *sec9-4* strain (Fig. 4) suggest a key contribution of these proteins for molecular interactions of Scs2p with SNARE proteins. To test this in a direct way, we monitored Scs2p BiFC signals in strains where *OSH1-3* were either deleted (*Δosh1-3)* or *OSH3* was overexpressed. In wt cells the Scs2p-Scs2p BiFC signal localized to intracellular rings, presumably the ER, and bright plasma membrane patches (Fig. 5a, b left, arrowheads). These Scs2p-Scs2p plasma membrane BiFC patches were very faint in the *Δosh1-3* strain (Fig. 5a, b, left), whereas the Scs2p-Scs2p BiFC signal was enhanced by *OSH3* overexpression (Fig. 5a, c left). Furthermore, the Scs2p-Sec9p BiFC signal, which in wt cells localized primarily to the plasma membrane of buds, was abolished in the *Δosh1-3* strain, whereas it was both enhanced and extended to the mother cell plasma membrane by *OSH3* overexpression (Fig. 5a–c, middle, dotted line). The Sec22p-Sso1p BiFC signal was only slightly affected (19% reduction) by the loss of Osh1-3p, but was enhanced by overexpression of *OSH3* (Fig. 5a–c, the rightmost panels). These results confirm an important contribution of Osh1-3p for the molecular interactions of Scs2p with itself and Sec9p as well as for Sec22p-Sso1p interactions. In contrast to the *sec9-4* strain, the Scs2p-Scs2p and Sec22p-Sso1p BiFC interactions were not completely lost in the *Δosh1-3* strain. This could be explained by the wt Sec9p in the *Δosh1-3* strain and/or by the possibility that other FFAT motif containing proteins might be affected by the *sec9-4* mutation.

### Mammalian ORP2 modulates VAPA oligomerization

**Fig. 6:**
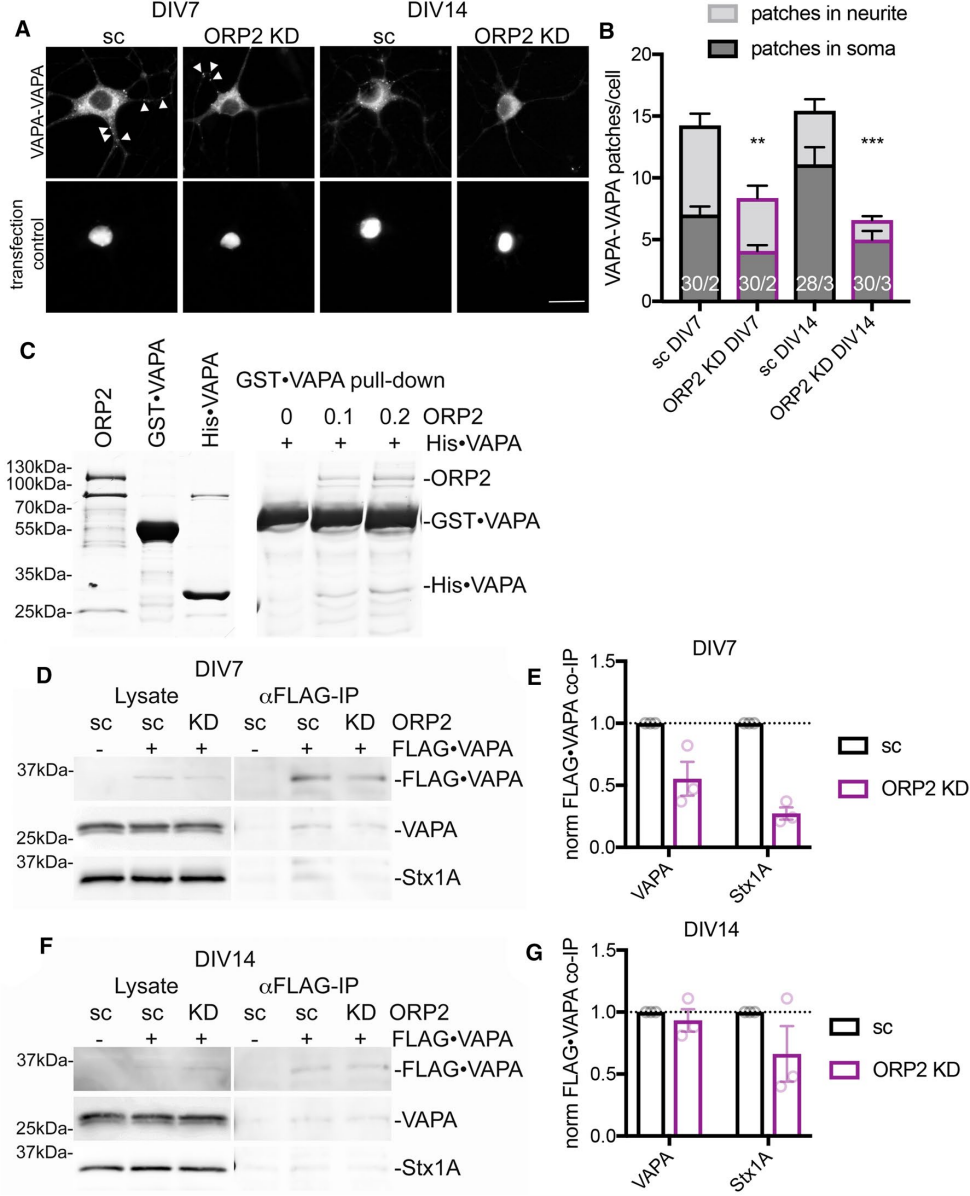
Mammalian ORP2 modulates VAPA oligomerization (**a**) ORP2 knock-down results in a partial loss of VAPA-VAPA BiFC interaction patches in mice hippocampal neurons at DIV 7 and DIV 14. Immunofluorescence imaging of wt mice hippocampal neurons with lentivirus-mediated knock-down of ORP2 (sc BL360, ORP2 KD BL1332), and Venus(N)·VAPA (BL1356) and Venus(C)·VAPA (BL1310) expression. Cells were infected at DIV 3 and fixed at DIV 7 or infected at DIV 7 and fixed at DIV 14. Scale bar, 20 µm. **b** Quantification of VAPA-VAPA BiFC interaction patches in (**a**). VAPA-VAPA BiFC clusters were manually counted in a minimum of 28 cells from 2–3 cultures. Shown is mean ± SEM, **p < 0.01, ***p < 0.001, number of cells/independent cultures. **c** ORP2 promotes VAPA oligomerization in vitro. 1 µM GST·VAPA(ΔTM) bound on Gluthatione-agarose was mixed with 0.4 µM His·VAPA(ΔTM) and increasing amounts (0, 0.1 and 0.2 µM, indicated above) of pFOLD·ORP2 and incubated at room temperature for 1 h. Bound proteins were separated by SDS–PAGE and stained with Coomassie blue. **d** Stx1A and VAPA association with FLAG·VAPA is largely abolished by ORP2 knock-down in DIV 7 neurons. Mice hippocampal neurons were infected with shRNA (sc BL360, ORP2 KD BL1332) and FLAG·VAPA (BL1466, -BL140) constructs at DIV 3. Cells were lysed at DIV 7, and lysates were subjected to anti-FLAG immunoprecipitations. Immunoprecipitates and lysates were analyzed by Western blotting with anti-VAPA and -Stx1A antibodies. **e** Quantification of three independent co-immunoprecipitations in (**d**). Shown is mean ± SEM. **f** VAPA association with FLAG·VAPA in unaffected by ORP2 knock-down in DIV 14 neurons. Experiment was performed as in (**d**), except that hippocampal neurons were infected at DIV 7 and lysed at DIV 14. **g** Quantification of three independent co-immunoprecipitations in (**f**). Shown is mean ± SEM

Similarly to Osh1-3p, several mammalian ORP proteins contain FFAT motifs that mediate interaction with the integral ER proteins VAPA and -B, the orthologues of yeast Scs2p [16, 17]. In wt mice hippocampal neurons, VAPA-VAPA BiFC interaction signals were observed throughout the neurons with patches in cell neurites and soma (Fig. 6a). These patches were reduced 1.7 and 2.3-fold in ORP2 KD cells (Supplementary Figure S3) when transduced at DIV 3 or DIV 7 and fixed at DIV 7 or DIV 14, respectively, suggesting that ORP2 is involved in VAPA oligomerization (Fig. 6a, b). On a side note, the evenly between cell soma and neurites distributed VAPA-VAPA BiFC signal patches at DIV 7 were predominantly observed in the cell soma at DIV 14 (Fig. 6a, arrowheads).

In HEK293 cells, VAPA-VAPA BiFC signals were detected at large structures in the cell. Overexpression of FFAT motif-containing ORPs resulted in an additional localization of the VAPA-VAPA BiFC signal to sites of ORP action, in the case of OSBP to the Golgi complex, ORP4L to the vimentin network, ORP1L to the late endosomes/lysosomes, ORP2 to lipid droplets, ORP3 to the ER network and ORP9L to perinuclear lines (Supplementary Figure S4A, arrow heads; and B). Of note, ORP10 lacking a FFAT motif did not influence the VAPA-VAPA BiFC signal or colocalize with it (Supplementary Figure S4A).

The observation that ORP2 knock-down or overexpression influences VAPA oligomerization could be a direct or indirect effect. We therefore performed in vitro assays with purified His_6_·pFOLD·ORP2, His_6_·VAPA and GST·VAPA to investigate whether purified ORP2 alone can promote VAPA oligomerization. Negligible co-purification of His_6_·VAPA with GST·VAPA was observed when they were incubated together without additional components (Fig. 6c, 0 µM ORP2). Addition of ORP2 promoted VAPA oligomerization (Fig. 6c, 0.1 or 0.2 µM ORP2). This result suggests that ORP2, containing a FFAT motif and being able to form tetramers [14], has the capacity to promote the VAPA oligomerization process by bridging the FFAT motif interacting sites in VAPA. This is consistent with the crystal structure reported for a VAP-FFAT complex, which contained two copies of both VAP and FFAT motif, with the FFAT motifs acting as bridges between the VAPs, as residues from each FFAT bound to both VAPs [17].

As the yeast ortholog of VAPA, Scs2p, oligomerizes with itself and interacts with the Q-SNARE Sec9p in an Oshp dependent manner (Fig. 5), we wanted to validate if ORP2 has similar effects on the VAPA interactions in the mammalian in vivo environment. We performed FLAG·VAPA immunoprecipitations in mouse hippocampal neurons in combination with ORP2 knock-down and examined the FLAG·VAPA interactions to endogenous VAPA and the Q-SNARE Stx1A. To be able to distinguish the FLAG·VAPA from the endogenous VAPA, we included a 10 kDa linker in the construct. FLAG·VAPA associated with endogenous VAPA and Stx1A in control neurons at DIV 7 and DIV 14 (Fig. 6d, f, sc lane). In ORP2 KD neurons at DIV 7 both VAPA and Stx1A association were reduced 45% and 73%, respectively (Fig. 6d, e), suggesting an ORP2 dependence of the VAPA self- and Q-SNARE interactions as shown in yeast. Intriguingly at DIV 14, VAPA interaction with itself and Stx1A were largely unaffected by ORP2 KD (Fig. 6f, g). This finding points to a more dominant role of ORP2 for these interactions at earlier time points (DIV 7), a time when VAPA-VAPA BiFC patches were observed in the neurites and the cell soma (Fig. 6a). At later timepoints (DIV 14), where VAPA-VAPA BiFC patches were mainly noted in the cell soma, ORP2 appears dispensable.

### Knock-down of ORP2 in younger neurons increases cell death and reduces dendrite length and synapse number

**Fig. 7:**
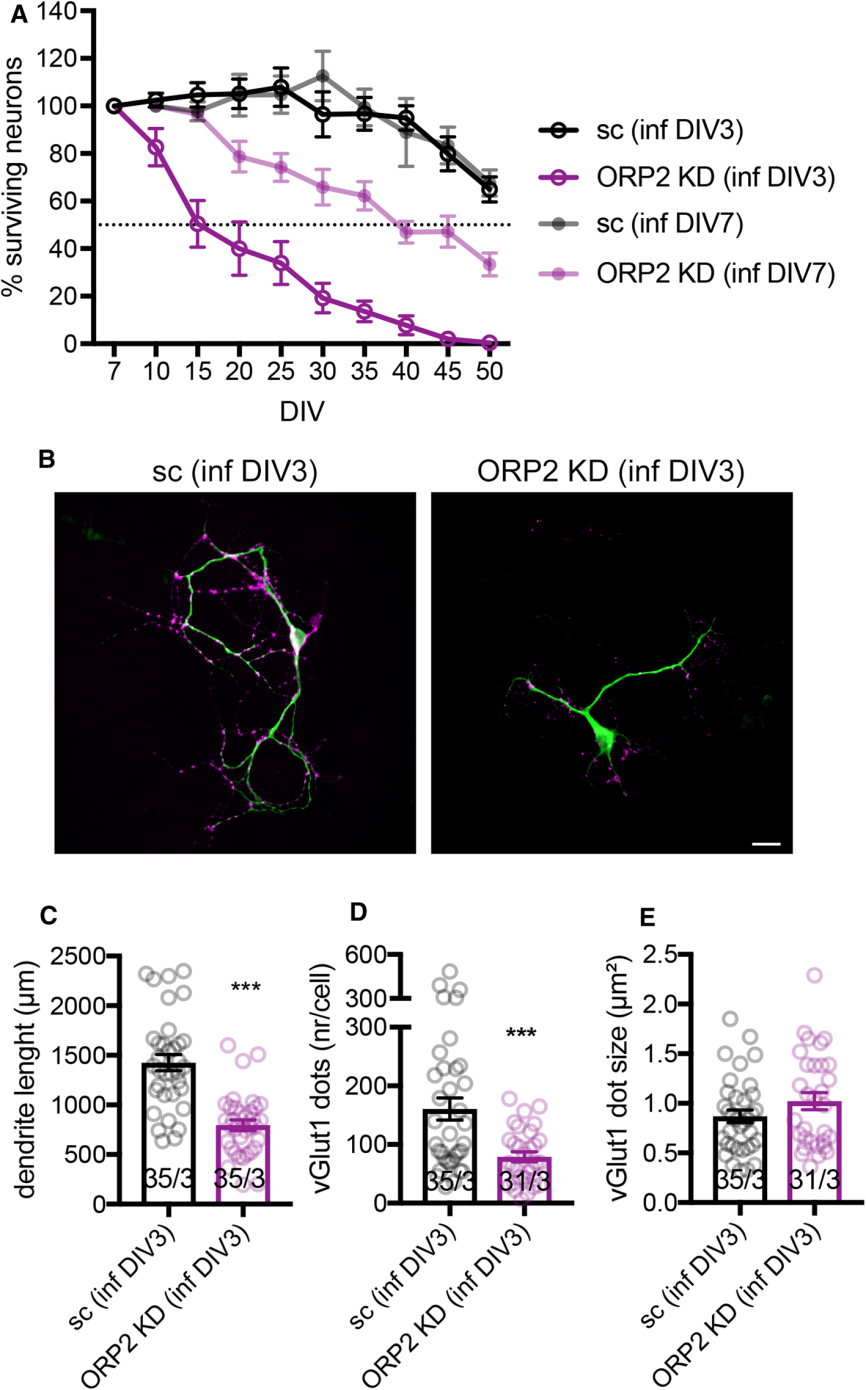
Knock-down of ORP2 in younger neurons reduces cell survival. **a** Knock-down at DIV 3, but not DIV 7, reduces cell survival. Summary graph of surviving neurons with ORP2 knock-down at DIV 3 or DIV 7 (sc BL360, ORP2 KD BL1332, percentage normalized to the number of infected neurons at DIV 5 or DIV 10, respectively). 40–60 images at each time point (± one day) were analyzed from two–three independent cultures. Shown is mean ± SEM. **b** ORP2 knock-down at DIV 3 alters neuronal cell morphology. Immunofluorescence imaging of wt mice hippocampal neurons with lentivirus-based knock-down of ORP2 at DIV 3 (sc BL360, ORP2 KD BL1332). Cells were fixed at DIV 14 and immunofluorescence staining against vGlut1 (magenta) and MAP2 (green) performed. Scale bar, 40 µm. **c** Quantification of dendrite length per cell (stained with MAP2) in (**b**). **d** and **e** Quantification of vGlut1 dot number and size per cell in (**b**). Shown is mean ± SEM, ***p < 0.001, number of cells/independent cultures

The different importance of ORP2 in ‘younger’ versus ‘older’ neurons was further examined by monitoring cell survival. While ORP2 knock-down at DIV 7 reduced neuronal survival only slightly, knock-down at DIV 3 resulted in 50% cells death after 15 days in culture (Fig. 7a). The surviving neurons at DIV 14 were characterized by a reduced dendritic length (MAP2, shown in green) and smaller synapse number (vGlut1, shown in magenta) compared to wt neurons (Fig. 7b–e), suggesting a positive role of ORP2 for neurite outgrowth. Knock-down of ORP2 at DIV 7 had no effect on neuronal morphology at DIV 14 (Supplementary Figure S5), confirming the major role of ORP2 for cell development at early stages.

### The Osh proteins negatively regulate exocytosis

**Fig. 8:**
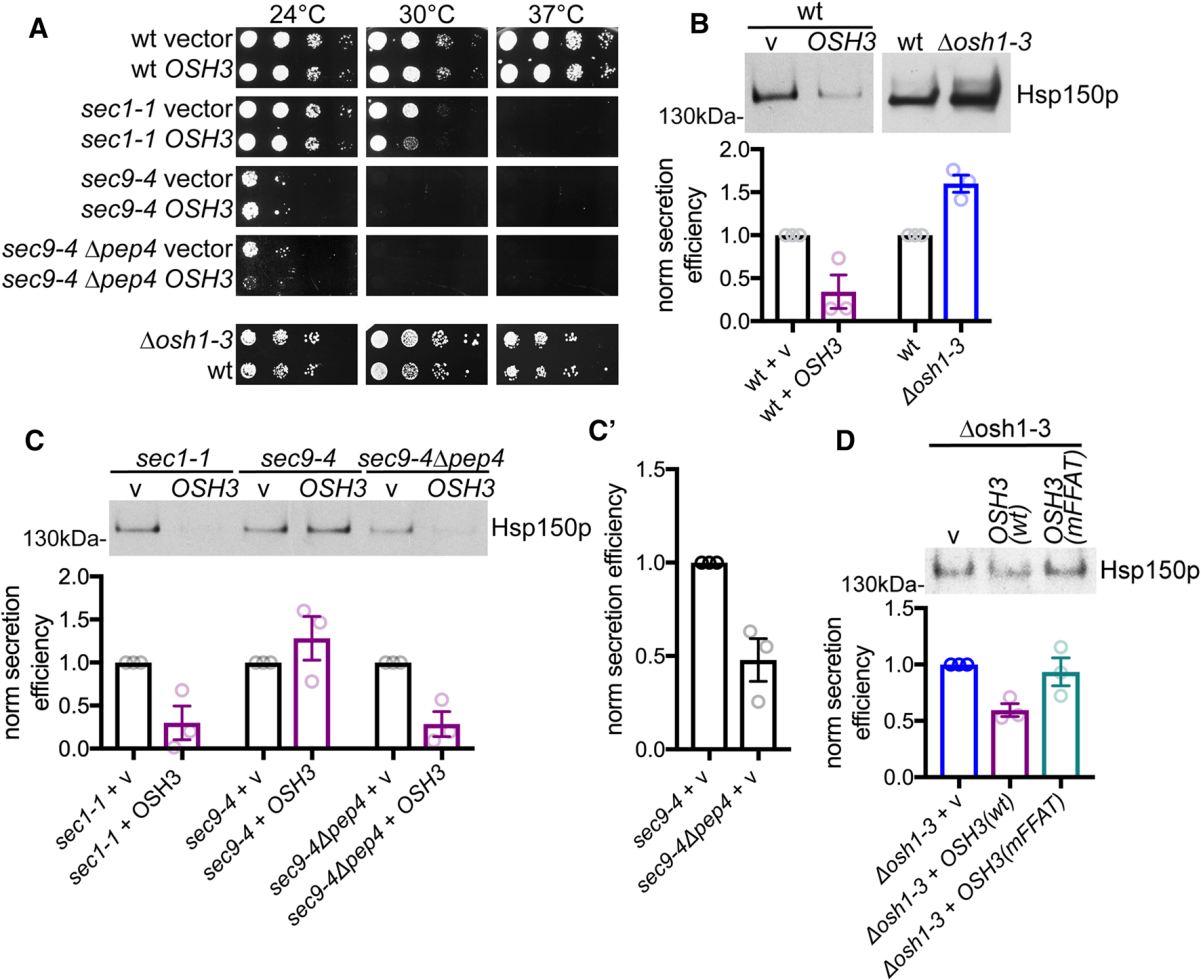
The Osh proteins negatively regulate exocytosis. **a** Overexpression of *OSH3* results in an enhanced temperature-sensitive growth phenotype in *sec1-1* and *sec9-4 Δpep4* cells. The growth of a serial tenfold dilution series was scored at the indicated temperatures in wt (Y1), *sec1-1* (Y15), *sec9-4* (Y6) and *sec9-4 Δpep4* (Y39) cells transformed either with an empty vector (1356) or YFP(N)·Osh3p (1364); and in the bottom panel wt (Y2) and *Δosh1-3* (Y36) cells. **b** Overexpression of *OSH3* results in reduced secretion of Hsp150, while *Δosh1-3* yeast cells display enhanced secretion of Hsp150p. Wt (Y1) cells transformed either with an empty vector (1356) or YFP(N)·Osh3p (1364) (left panel) and wt (Y2) or *Δosh1-3* (Y36) cells (right panel) were grown at 24 °C until they reached an A600 of 0.3. The cells were washed, resuspended in fresh medium to A600 of 0.3, and shifted to 34 °C. Samples were collected after 2 h. Cells were removed by centrifugation and the amount of Hsp150p in a 30 μl aliquot of the growth medium was quantified by western blotting. Quantification of three independent experiments shown in. Shown are the means of secretion efficiencies ± SEM, normalized to the corresponding wt control. **c** Reduced secretion of Hsp150 protein upon *OSH3* overexpression in *sec1-1* and *sec9-4* mutants. Experiments were performed as in (**b**) using yeast cells as in (**a**). Shown are the means of secretion efficiencies ± SEM, normalized to the corresponding vector control. **c’** *PEP4* deletion in the *sec9-4* mutant results in reduced secretion of Hsp150. Quantification of the secretion efficiency of *sec9-4* + v vs. *sec9-4 Δpep4* + v cells shown in (**c**). Shown are the means of secretion ± SEM efficiencies, normalized to *sec9-4* + v. **d** Scs2p binding deficient *OSH3* does not cause an Hsp150p secretion defect*. *Experiment was essentially performed as in (**b**) using* Δosh1-3* (Y36) cells expressing an empty vector (1356), YFP(N)·Osh3p (1364) or YFP(N)·Osh3p(mFFAT) (1389) and sample collection after 1 h incubation at 37 °C. Shown are means of normalized secretion efficiencies ± SEM

Next, we asked whether there is a functional link between Osh proteins and secretion. Overexpression of *OSH3* was toxic, resulting in enhanced temperature sensitivity, in two different strains with deficient secretion: *sec1-1* [62] and *sec9-4 Δpep4* mutant cells (Fig. 8a). This toxic effect of Osh3p required a lesion in secretion, as it was not seen in wt or in *Δosh1-3* cells (Fig. 8a). It was also not observed in *sec9-4* mutants, likely due to Osh3p degradation in this strain (Figs. 2a and 8a).

To obtain a more quantitative measure of how Osh3p affects protein secretion, we assayed secretion of the heat shock protein Hsp150p. Overexpression of *OSH3* in wt cells reduced Hsp150p secretion by 66%, while *OSH1-3* deletion (*Δosh1-3*) enhanced Hsp150p secretion by 60% (Fig. 8b). In line, in the secretion deficient *sec1-1* and *sec9-4 Δpep4* strains*,* Hsp150p secretion was further reduced by *OSH3* overexpression (Fig. 8c). Again, *OSH3* overexpression had no effect in the *sec9-4* strain, likely because of Osh3p degradation in this strain (Figs. 2a and 8c). Importantly, loss of *PEP4* in the *sec9-4* strain reduced secretion of Hsp150p by 52% (Fig. 8c and c’ compare *sec9-4* + v with *sec9-4 Δpep4* + v), suggesting that vacuolar degradation of Osh proteins in *sec9-4* cells enhances secretion, and is in line with the observed enhanced secretion in *Δosh1-3* cells (Fig. 8b). Finally, while reintroduction of Osh3p (wt) to the *Δosh1-3* stain reduced Hsp150p secretion as expected, the Scs2p binding-deficient Osh3p(mFFAT) had no significant effect on secretion (Fig. 8d), suggesting that Scs2p binding is needed for the toxic secretion effect of Osh3p.

## Discussion

In yeast the cortical ER is extensively tethered to the plasma membrane, with approximately 1000 contact patches of the two membrane compartments operating at a given time point, and 20–45% of the PM area participating in the contacts [63, 64]. Conceivably, vesicle transport to and from the PM needs to be coordinated with the ER-PM contacts (MCSs), which could form a barrier for transport vehicles. Spatial coordination of endocytic events and eisosomes with the cortical ER has been described [40–42]. Similarly, co-regulation of exocytic events with MCSs has been proposed in fission yeast [65]. Yet, the molecular mechanisms underlying this coordination are unknown.

Both MCS formation and vesicle transport involve specific tethering of the participating membranes. Three yeast Osh proteins (Osh1-3p) are anchored to the endoplasmic reticulum (ER) via interactions with the membrane protein Scs2p, the yeast orthologue of mammalian VAPs, and Osh2/3p-Scs2p complexes are located at ER-PM MCSs [13, 66]. We initially observed that a mutation in yeast *SEC9* (a secretory Q-SNARE) resulted in a loss of complexes between Osh1-3p and Scs2p, due to destabilization of all of Osh1-3p. Intriguingly, other complexes that were not known to contain Osh proteins were also lost: Scs2p with itself and with secretory SNAREs. Disruption of the vacuolar aspartyl protease *PEP4* rescued all these phenotypes and the Osh protein levels, suggesting that Osh1-3p are degraded in the yeast vacuole.

VAPs/Scs2p interact with a large number of intracellular proteins and have been implicated in the regulation of a wide range of cellular processes, including membrane trafficking, lipid transport and metabolism, the unfolded protein response and microtubule organization [67]. The mammalian orthologues of Ssc2p, VAPA and -B, form homo- and heterodimers [68, 69], and VAPA has been crystallized as a homodimer in which each subunit binds to the FFAT motif containing peptide of an ORP protein [17]. Also, mammalian OSBP/ORP4L [6, 70] and ORP3 [21] have been reported to form dimers while ORP2 has been shown to form tetramers [14], consistent with the idea that oligomeric ORP/Osh proteins could act as cores around which a complex containing multiple copies of VAP/Scs2p forms. We show for the first time that VAPA and Scs2p form reversible complexes that contain dimers or possibly larger oligomers of these proteins, upon binding to ORP2 or Osh1-3p, respectively. We envision that this could be a common effect of binding to any of the ~ 50 proteins with strong FFAT or FFAT-like motifs [67].

Complexes surrounding VAP/Scs2p-ORP/Osh proteins include the SNAREs Scs22p (ER) and Stx1A/Sso1p (plasma membrane). Mammalian Sec22 and Stx1 have been proposed to form a complex at ER-PM contacts that is non-fusogenic, and is essential for neurite outgrowth, possibly by promoting the delivery of lipid to the growing neurite plasma membrane [39]. Interestingly, the authors also demonstrated that both Sso1p and Sec22p co-immunoprecipitated with Osh2p and Osh3p in yeast, supporting an intimate connection between these SNAREs and the Osh at MCSs. In line, knock-down of ORP2, the main ORP delivering cholesterol to the plasma membrane [14], in ‘younger’ neurons (DIV 3) decreased the dendritic length and number of synapses per neuron. ORP2 knock-down at a later time point (DIV 7), when VAPA oligomers are less pronounced in the neurites and unaffected by ORP2 knock-down, had no effect on neuronal morphology, indicating the involvement of other proteins at later time points. In agreement, neuron survival was especially reduced by early knock-down of ORP2 (DIV 3).

Osh3p was shown, by pull-down assays, to interact physically with Sec9p. Likewise, nine of the twelve human ORPs were in close proximity of SNAP-25, and at least one (ORP2) bound directly to SNAP-25 and was degraded (along with ORP1L) in SNAP-25 KO mice hippocampal neurons. Collated with the observations of Petkovic et al. [39], the present findings indicate that not only Sec22 and Stx1A/Sso1p, but also SNAP-25/Sec9p, participate in interactions with the ER-PM MCS ORP/Osh proteins, and that SNAP-25/Sec9p may in fact play a key role in the interactions of the SNAREs with the ORP/Osh-based MCS protein machinery.

Interestingly, our experiments performed on *sec9-4 Δpep4* cells suggest that the Osh proteins act in a regulatory pathway upstream of secretory SNAREs. In line, the observed enhancement of Hsp150p secretion in *Δosh1-3* cells and its inhibition by overexpression of Osh3p suggest that the ER-PM MCSs or more specifically, the Oshp at these sites, exert a negative regulatory action on secretion. Importantly, an Osh3p mutant with the FFAT motif inactivated and thus incapable of binding to Scs2p at ER membranes had no effect on secretion, suggesting a functional interplay of Osh3p with the secretory machinery at ER-PM contacts. We envision that the ORP/Osh proteins, possibly in concert with other yet unknown protein components, might regulate the distinct PM regions where ER-PM MCS and exocytotic sites are in close proximity by assigning the fate of PM associated Stx1A/Sso1p and SNAP-25/Sec9p either to ER-PM MCS or secretory vesicle fusion. The precise regulatory mode explaining how these two processes, requiring partially the same proteins (possibly in different subcomplexes), do not interfere with each other will need further investigation, with the ORP/Osh proteins being likely candidates to be involved.

In conclusion, the present data demonstrate a novel functional coordination between the machineries operating at ER-PM membrane contact sites and exocytic membrane fusion. Our findings also suggest that certain SNAREs, SNAP-25/Sec9p in particular, execute a novel function in MCS formation.

### Electronic supplementary material

Below is the link to the electronic supplementary material.

Supplementary file1 (DOCX 59 kb)

Supplementary file2 Supplementary Figure 1. Mutants defective in exocytic SNARE complex assembly or disassembly do not affect Oshp-Scs2p BiFC interaction. Live cell imaging of indicated vegetatively grown haploid cells (wt, Y1; sec4-8, Y19; sec18-1, Y13; Δmso1, Y14; sec1-1, Y15; Δsro7, Y17) expressing YFP(N)·Osh1p (1362, A), YFP(N)·Osh2p (1363, B) or YFP(N)·Osh3p (1364, C) in combination with YFP(C)·Scs2p (1360). BiFC interactions were monitored by fluorescence microscopy in in a minimum of 28 cells grown at 24°C. The data represent mean ± SEM. Scale bar, 5 µm(TIF 15707 kb).

Supplementary file3 Supplementary Figure 2. The plasma membrane SNAREs Sec9p and Sso1/2p co-immunoprecipitate with Osh3p, while Osh1p associates with Snc1/2p. (A). *Indicated yeast* cells (wt, Y1; sec9-4, Y6; sso2-1 Δsso1, Y7; Δscn1, Y12; sec4-8, Y19) expressing either empty vector (-; 1356) or YFP(N)·HA·Osh3p (1477) were grown until OD600 = 1, lysed, and subjected to anti-HA immunoprecipitations. Immunoprecipitates and lysates were analyzed by Western blotting with anti-HA, -Sec9p, and -Sso1/2p antibodies. (B) Quantification of three independent experiments of immunoprecipitations shown in (A). The data represent mean ± SEM. (C)* Wt yeast* cells (Y1) expressing either empty vector (-; 1356) or YFP(N)·HA·Osh1p (1513) were grown until OD600 = 1, lysed, and subjected to anti-HA immunoprecipitations. Immunoprecipitates and lysates were analyzed by Western blotting with anti-HA, -Sso1/2p, and -Snc1p antibodies(TIF 24549 kb).

Supplementary file4 Supplementary Figure 3. ORP2 Knock-down efficiency in mice hippocampal neurons. (A) SDS lysates of lentivirus-based ORP2 KD in mice hippocampal neurons at DIV 7. Cells were transduced with scrambled shRNA (sc, BL360) or shRNA against ORP2 (ORP2 KD, BL1332) at DIV 3 and lysed at DIV 7. SDS lysates were analyzed by Western blotting and detection and anti-ORP2 and -β-Tubulin antibodies. (B) Quantification of ORP2 KD efficiency in three independed cultures as in (A). (C) SDS lysates of lentivirus-based ORP2 KD in mice hippocampal neurons at DIV 14. Cells were transduced with scrambled shRNA (sc, BL360) or shRNA against ORP2 (ORP2 KD, BL1332) at DIV 7 and lysed at DIV 14. SDS lysates were analyzed by Western blotting and detection and anti-ORP2 and -β-Tubulin antibodies. (D) Quantification of ORP2 KD efficiency in three independed cultures as in (C)(TIF 24548 kb).

Supplementary file5 Supplementary Figure 4. Overexpression of certain ORPs causes VAPA oligomer re-localization in vivo. (A) Fluorescence imaging of HEK293 cells transfected with plasmids expressing Venus(N)·VAPA (1560) and Venus(C)·VAPA (1223) in combination with indicated mCherry·ORP constructs (-, 1140; OSBP, 1274; ORP4L, 1276; ORP1L, 1269; ORP2, 1271; ORP3, 1275; ORP9L, 1277; ORP10, 1161). Arrowheads point to VAPA oligomers localizing to the Golgi complex (OSBP), Vimentin intermediate filament (ORP4L), late endosomes (ORP1L) and lipid droplets (ORP2). Scale bar 10µm. (B) Fluorescence imaging as in (A) with additional staining with anti-PDI antibody for ER, anti-LAMP1 antibody for late endosomes, LipidTox for lipid droplets, anti-Vimentin antibody for Vimentin intermediate filament and anti-GM130 antibody for Golgi complex. Scale bar, 10 µm(TIF 24553 kb).

Supplementary file6 Supplementary Figure 5. ORP2 knock-down at DIV 7 does not alter neuronal cell morphology. (A) Immunofluorescence imaging of wt mice hippocampal neurons with lentivirus-based knock-down of ORP2 at DIV 7 (sc BL360, ORP2 KD BL1332). Cells were fixed at DIV 14 and immunofluorescence staining against vGlut1 (magenta) and MAP2 (green) performed. Scale bar 40µm. (B) Quantification of dendrite length per cell (stained with MAP2) in (A). (C and D) Quantification of vGlut1 dot number and size per cell in (A). Shown is mean +/-SEM, number of cells/independent cultures(TIF 24549 kb).

## Data Availability

The authors confirm that the data supporting the findings of this study are available within the article and its supplementary materials. Additional data and materials of this study are available from the corresponding authors, M.W.-B. and C.R., upon request.

## Abbreviations

BiFC: Bimolecular fluorescence complementation
ER: Endoplasmic reticulum
FFAT: Two phenylalanines in an acidic tract
KD: Knock-down
KO: Knock-out
MCS: Membrane contact site
ORP: OSBP-related protein
OSBP: Oxysterol-binding protein
Osh: OSBP homolog (in yeast)
PM: Plasma membrane
SNARE: Soluble n-ethylmaleimide sensitive factor attachment protein receptor
VAMP: Vesicle-associated membrane protein
VAP: VAMP-associated protein
wt: Wild type

## References

[1] Taylor FR, Saucier SE, Shown EP, Parish EJ, Kandutsch AA (1984). Correlation between oxysterol binding to a cytosolic binding protein and potency in the repression of hydroxymethylglutaryl coenzyme A reductase. J Biol Chem.

[2] Dawson PA, Ridgway ND, Slaughter CA, Brown MS, Goldstein JL (1989). cDNA cloning and expression of oxysterol-binding protein, an oligomer with a potential leucine zipper. J Biol Chem.

[3] Maeda K, Anand K, Chiapparino A, Kumar A, Poletto M, Kaksonen M, Gavin AC (2013). Interactome map uncovers phosphatidylserine transport by oxysterol-binding proteins. Nature.

[4] Wang PY, Weng J, Anderson RG (2005). OSBP is a cholesterol-regulated scaffolding protein in control of ERK 1/2 activation. Science.

[5] Mesmin B, Bigay J, Moser von Filseck J, Lacas-Gervais S, Drin G, Antonny B (2013). A four-step cycle driven by PI(4)P hydrolysis directs sterol/PI(4)P exchange by the ER-Golgi tether OSBP. Cell.

[6] Wyles JP, Perry RJ, Ridgway ND (2007). Characterization of the sterol-binding domain of oxysterol-binding protein (OSBP)-related protein 4 reveals a novel role in vimentin organization. Exp Cell Res.

[7] Vihervaara T, Uronen RL, Wohlfahrt G, Bjorkhem I, Ikonen E, Olkkonen VM (2011). Sterol binding by OSBP-related protein 1L regulates late endosome motility and function. Cell Mol Life Sci.

[8] Suchanek M, Hynynen R, Wohlfahrt G, Lehto M, Johansson M, Saarinen H, Radzikowska A, Thiele C, Olkkonen VM (2007). The mammalian oxysterol-binding protein-related proteins (ORPs) bind 25-hydroxycholesterol in an evolutionarily conserved pocket. Biochem J.

[9] Hynynen R, Suchanek M, Spandl J, Back N, Thiele C, Olkkonen VM (2009). OSBP-related protein 2 is a sterol receptor on lipid droplets that regulates the metabolism of neutral lipids. J Lipid Res.

[10] Im YJ, Raychaudhuri S, Prinz WA, Hurley JH (2005). Structural mechanism for sterol sensing and transport by OSBP-related proteins. Nature.

[11] Raychaudhuri S, Im YJ, Hurley JH, Prinz WA (2006). Nonvesicular sterol movement from plasma membrane to ER requires oxysterol-binding protein-related proteins and phosphoinositides. J Cell Biol.

[12] de Saint-Jean M, Delfosse V, Douguet D, Chicanne G, Payrastre B, Bourguet W, Antonny B, Drin G (2011). Osh4p exchanges sterols for phosphatidylinositol 4-phosphate between lipid bilayers. J Cell Biol.

[13] Schulz TA, Choi MG, Raychaudhuri S, Mears JA, Ghirlando R, Hinshaw JE, Prinz WA (2009). Lipid-regulated sterol transfer between closely apposed membranes by oxysterol-binding protein homologues. J Cell Biol.

[14] Wang H, Ma Q, Qi Y, Dong J, Du X, Rae J, Wang J, Wu WF, Brown AJ, Parton RG, Wu JW, Yang H (2019). ORP2 delivers cholesterol to the plasma membrane in exchange for phosphatidylinositol 4,5-bisphosphate (PI(4,5)P2). Mol Cell.

[15] Ghai R, Du X, Wang H, Dong J, Ferguson C, Brown AJ, Parton RG, Wu JW, Yang H (2017). ORP5 and ORP8 bind phosphatidylinositol-4,5-biphosphate (PtdIns(4,5)P 2) and regulate its level at the plasma membrane. Nat Commun.

[16] Loewen CJ, Roy A, Levine TP (2003). A conserved ER targeting motif in three families of lipid binding proteins and in Opi1p binds VAP. EMBO J.

[17] Kaiser SE, Brickner JH, Reilein AR, Fenn TD, Walter P, Brunger AT (2005). Structural basis of FFAT motif-mediated ER targeting. Structure.

[18] Levine TP, Munro S (1998). The pleckstrin homology domain of oxysterol-binding protein recognises a determinant specific to Golgi membranes. Curr Biol.

[19] Levine TP, Munro S (2002). Targeting of Golgi-specific pleckstrin homology domains involves both PtdIns 4-kinase-dependent and -independent components. Curr Biol.

[20] Johansson M, Lehto M, Tanhuanpaa K, Cover TL, Olkkonen VM (2005). The oxysterol-binding protein homologue ORP1L interacts with Rab7 and alters functional properties of late endocytic compartments. Mol Biol Cell.

[21] Lehto M, Hynynen R, Karjalainen K, Kuismanen E, Hyvarinen K, Olkkonen VM (2005). Targeting of OSBP-related protein 3 (ORP3) to endoplasmic reticulum and plasma membrane is controlled by multiple determinants. Exp Cell Res.

[22] Kvam E, Goldfarb DS (2004). Nvj1p is the outer-nuclear-membrane receptor for oxysterol-binding protein homolog Osh1p in *Saccharomyces cerevisiae*. J Cell Sci.

[23] Levine TP, Munro S (2001). Dual targeting of Osh1p, a yeast homologue of oxysterol-binding protein, to both the Golgi and the nucleus-vacuole junction. Mol Biol Cell.

[24] Rocha N, Kuijl C, van der Kant R, Janssen L, Houben D, Janssen H, Zwart W, Neefjes J (2009). Cholesterol sensor ORP1L contacts the ER protein VAP to control Rab7-RILP-p150 Glued and late endosome positioning. J Cell Biol.

[25] Stefan CJ, Manford AG, Emr SD (2013). ER-PM connections: sites of information transfer and inter-organelle communication. Curr Opin Cell Biol.

[26] Stefan CJ, Manford AG, Baird D, Yamada-Hanff J, Mao Y, Emr SD (2011). Osh proteins regulate phosphoinositide metabolism at ER-plasma membrane contact sites. Cell.

[27] Aalto MK, Ronne H, Keranen S (1993). Yeast syntaxins Sso1p and Sso2p belong to a family of related membrane proteins that function in vesicular transport. EMBO J.

[28] Jahn R, Scheller RH (2006). SNAREs–engines for membrane fusion. Nat Rev Mol Cell Biol.

[29] van den Bogaart G, Holt MG, Bunt G, Riedel D, Wouters FS, Jahn R (2010). One SNARE complex is sufficient for membrane fusion. Nat Struct Mol Biol.

[30] Sutton RB, Fasshauer D, Jahn R, Brunger AT (1998). Crystal structure of a SNARE complex involved in synaptic exocytosis at 2.4 A resolution. Nature.

[31] Strop P, Kaiser SE, Vrljic M, Brunger AT (2008). The structure of the yeast plasma membrane SNARE complex reveals destabilizing water-filled cavities. J Biol Chem.

[32] He B, Guo W (2009). The exocyst complex in polarized exocytosis. Curr Opin Cell Biol.

[33] Jahn R, Lang T, Sudhof TC (2003). Membrane fusion. Cell.

[34] Jahn R, Fasshauer D (2012). Molecular machines governing exocytosis of synaptic vesicles. Nature.

[35] Novick P, Guo W (2002). Ras family therapy: Rab, Rho and Ral talk to the exocyst. Trends Cell Biol.

[36] Rizo J, Sudhof TC (2012). The membrane fusion enigma: SNAREs, Sec1/Munc18 proteins, and their accomplices—guilty as charged?. Annu Rev Cell Dev Biol.

[37] Toonen RF, Verhage M (2003). Vesicle trafficking: pleasure and pain from SM genes. Trends Cell Biol.

[38] Sudhof TC, Rothman JE (2009). Membrane fusion: grappling with SNARE and SM proteins. Science.

[39] Petkovic M, Jemaiel A, Daste F, Specht CG, Izeddin I, Vorkel D, Verbavatz JM, Darzacq X, Triller A, Pfenninger KH, Tareste D, Jackson CL, Galli T (2014). The SNARE Sec22b has a non-fusogenic function in plasma membrane expansion. Nat Cell Biol.

[40] Stradalova V, Blazikova M, Grossmann G, Opekarova M, Tanner W, Malinsky J (2012). Distribution of cortical endoplasmic reticulum determines positioning of endocytic events in yeast plasma membrane. PLoS ONE.

[41] Deutsch E, Weigel AV, Akin EJ, Fox P, Hansen G, Haberkorn CJ, Loftus R, Krapf D, Tamkun MM (2012). Kv2.1 cell surface clusters are insertion platforms for ion channel delivery to the plasma membrane. Mol Biol Cell.

[42] Fox PD, Haberkorn CJ, Akin EJ, Seel PJ, Krapf D, Tamkun MM (2015). Induction of stable ER-plasma-membrane junctions by Kv2.1 potassium channels. J Cell Sci.

[43] Kozminski KG, Alfaro G, Dighe S, Beh CT (2006). Homologues of oxysterol-binding proteins affect Cdc42p- and Rho1p-mediated cell polarization in *Saccharomyces cerevisiae*. Traffic.

[44] Alfaro G, Johansen J, Dighe SA, Duamel G, Kozminski KG, Beh CT (2011). The sterol-binding protein Kes1/Osh4p is a regulator of polarized exocytosis. Traffic.

[45] Ling Y, Hayano S, Novick P (2014). Osh4p is needed to reduce the level of phosphatidylinositol-4-phosphate on secretory vesicles as they mature. Mol Biol Cell.

[46] Sherman F (1991). Getting started with yeast. Methods Enzymol.

[47] Lois C, Hong EJ, Pease S, Brown EJ, Baltimore D (2002). Germline transmission and tissue-specific expression of transgenes delivered by lentiviral vectors. Science.

[48] Kim JH, Lee SR, Li LH, Park HJ, Park JH, Lee KY, Kim MK, Shin BA, Choi SY (2011). High cleavage efficiency of a 2A peptide derived from porcine teschovirus-1 in human cell lines, zebrafish and mice. PLoS ONE.

[49] Vardar G, Chang S, Arancillo M, Wu YJ, Trimbuch T, Rosenmund C (2016). Distinct functions of syntaxin-1 in neuronal maintenance, synaptic vesicle docking, and fusion in mouse neurons. J Neurosci.

[50] Knop M, Miller KJ, Mazza M, Feng D, Weber M, Keranen S, Jantti J (2005). Molecular interactions position Mso1p, a novel PTB domain homologue, in the interface of the exocyst complex and the exocytic SNARE machinery in yeast. Mol Biol Cell.

[51] Laitinen S, Lehto M, Lehtonen S, Hyvarinen K, Heino S, Lehtonen E, Ehnholm C, Ikonen E, Olkkonen VM (2002). ORP2, a homolog of oxysterol binding protein, regulates cellular cholesterol metabolism. J Lipid Res.

[52] Jantti J, Aalto MK, Oyen M, Sundqvist L, Keranen S, Ronne H (2002). Characterization of temperature-sensitive mutations in the yeast syntaxin 1 homologues Sso1p and Sso2p, and evidence of a distinct function for Sso1p in sporulation. J Cell Sci.

[53] Russo P, Kalkkinen N, Sareneva H, Paakkola J, Makarow M (1992). A heat shock gene from *Saccharomyces cerevisiae* encoding a secretory glycoprotein. Proc Natl Acad Sci U S A.

[54] Novick P, Field C, Schekman R (1980). Identification of 23 complementation groups required for post-translational events in the yeast secretory pathway. Cell.

[55] Kerppola TK (2008). Bimolecular fluorescence complementation (BiFC) analysis as a probe of protein interactions in living cells. Annu Rev Biophys.

[56] Weber-Boyvat M, Li S, Skarp KP, Olkkonen VM, Yan D, Jantti J (2015). Bimolecular fluorescence complementation (BiFC) technique in yeast *Saccharomyces cerevisiae* and mammalian cells. Methods Mol Biol.

[57] Manik MK, Yang H, Tong J, Im YJ (2017). Structure of yeast OSBP-related protein Osh1 reveals key determinants for lipid transport and protein targeting at the nucleus-vacuole junction. Structure.

[58] Rossi G, Salminen A, Rice LM, Brunger AT, Brennwald P (1997). Analysis of a yeast SNARE complex reveals remarkable similarity to the neuronal SNARE complex and a novel function for the C terminus of the SNAP-25 homolog, Sec9. J Biol Chem.

[59] Brennwald P, Kearns B, Champion K, Keranen S, Bankaitis V, Novick P (1994). Sec9 is a SNAP-25-Like component of a yeast snare complex that may be the effector of Sec4 function in exocytosis. Cell.

[60] Rossi G, Watson K, Demonch M, Temple B, Brennwald P (2015). In vitro reconstitution of Rab GTPase-dependent vesicle clustering by the yeast lethal giant larvae/tomosyn homolog, Sro7. J Biol Chem.

[61] Krick R, Muehe Y, Prick T, Bremer S, Schlotterhose P, Eskelinen EL, Millen J, Goldfarb DS, Thumm M (2008). Piecemeal microautophagy of the nucleus requires the core macroautophagy genes. Mol Biol Cell.

[62] Novick P, Schekman R (1979). Secretion and cell-surface growth are blocked in a temperature-sensitive mutant of *Saccharomyces cerevisiae*. Proc Natl Acad Sci U S A.

[63] Loewen CJ, Levine TP (2005). A highly conserved binding site in vesicle-associated membrane protein-associated protein (VAP) for the FFAT motif of lipid-binding proteins. J Biol Chem.

[64] West M, Zurek N, Hoenger A, Voeltz GK (2011). A 3D analysis of yeast ER structure reveals how ER domains are organized by membrane curvature. J Cell Biol.

[65] Ng AYE, Ng AQE, Zhang D (2018). ER-PM contacts restrict exocytic sites for polarized morphogenesis. Curr Biol.

[66] Weber-Boyvat M, Kentala H, Peranen J, Olkkonen VM (2015). Ligand-dependent localization and function of ORP–VAP complexes at membrane contact sites. Cell Mol Life Sci.

[67] Murphy SE, Levine TP (2016). VAP, a versatile access point for the endoplasmic reticulum: review and analysis of FFAT-like motifs in the VAPome. Biochim Biophys Acta.

[68] Nishimura Y, Hayashi M, Inada H, Tanaka T (1999). Molecular cloning and characterization of mammalian homologues of vesicle-associated membrane protein-associated (VAMP-associated) proteins. Biochem Biophys Res Commun.

[69] Russ WP, Engelman DM (2000). The GxxxG motif: a framework for transmembrane helix-helix association. J Mol Biol.

[70] Ridgway ND, Dawson PA, Ho YK, Brown MS, Goldstein JL (1992). Translocation of oxysterol binding protein to Golgi apparatus triggered by ligand binding. J Cell Biol.

