# Supplementary material for: ORP/Osh mediate cross-talk between ER-plasma membrane contact site components and plasma membrane SNAREs
Source: Cell Mol Life Sci. 2020 Jul 30;78(4):1689–708. doi: 10.1007/s00018-020-03604-w (PMC7904734; doi:10.1007/s00018-020-03604-w)
Supplement: Supplementary file 1 — Supplementary file1 (DOCX 59 kb) [file 18_2020_3604_MOESM1_ESM.docx]

**Supplementary Table S1. Yeast strains**

| Name | Genotype | Source |
| --- | --- | --- |
| Y1  Y2  Y6  Y7  Y8  Y9  Y12  Y13  Y14  Y15  Y17  Y19  Y36  Y38  Y39 | MAT**a** *leu2-3,112 ura3-52*  *MATα leu2-3,112 ura3-52 his3Δ200 lys2-801 trp1Δ901 suc2Δ9*  MAT**a** *sec9-4 leu2-3,112 ura3-52*  *MAT****a*** *sso2-1 leu2-3,112 trp1-1 ura3-1 sso1::HIS3 ade2-1 his3-11,15 can1-100*  *MATα leu2 ura3 sso1::KanMX*  *MATα leu2 ura3 sso2::KanMX*  *MAT****a*** *his3 leu2 met15 ura3 snc1::kanMX*  *MATα sec18-1 leu2-3,112 trp1-289 ura3-52*  *MAT****a*** *leu2-3,112 ura3-52 mso1::hphMX4 GAL+*  *MAT****a*** *leu2-3,112 ura3-52 his4-619 sec1-1*  *MAT***a** *his3Δ, leu2Δ, met15Δ, ura3Δ, sro7::kanMX*  *MATα leu2-3,112 ura3-52 sec4-8*  *MATα leu2-3,112 ura3-52 his3Δ200 lys2-801 trp1Δ901 suc2Δ9 osh1::URA3 osh2::URA3 osh3::LYS2*  *MAT***a** *ura3-52 lys2-801 trp1del63 his3del200 leu2del1*  *MAT***a** *sec9-4 leu2-3,112 ura3-52 pep4::hphMX4* | P. Novick (NY179)  C. Beh (CBY1)  P. Novick (NY782)  H. Ronne (H604)  H. Ronne (H1398)  H. Ronne (H1400)  Euroscarf  R. Schekmann (mB12-16D)  Knop et al., 2005 (H2658)  J. Jäntti (H3019)  W. Guo (GY1334)  P. Novick (NY774)  C. Beh (CBY351)  M. Knop (EMS356-1)  This study |

**Supplementary Table S2. Plasmids**

| **Yeast Plasmids** | | | | | | | | | | | | |
| --- | --- | --- | --- | --- | --- | --- | --- | --- | --- | --- | --- | --- |
| Plasmid | Name | | Type | Yeast Promoter | | Insert | | | Marker | | Source | |
| 1356  1358  1360  1362  1363  1364  1365  1366  1367  1368  1389  1395  1399  1404  1468  1476  1477  1478  1513  1515  1516  1517  1525  1527  1530  1565  1566  1567  1572  1573  1574  1575  1577 | N-YN425ADH  N-YC426ADH  YEpYC-SCS2-U  YEpYN-OSH1-L  YEpYN-OSH2-L  YEpYN-OSH3-L  YEpYN-OSH4-L  YEpYN-OSH5-L  YEpYN-OSH6-L  YEpYN-OSH7-L  YEpYN-OSH3(mFFAT)-L  YEpYN-SSO1-L  YEpYN-Sec9-L  YEpYC-SEC9-U  N-YN(2HA)425ADH  YEpYN-2HA-OSH2-L  YEpYN-2HA-OSH3-L  YEpYC-3Flag-SCS2-U  YEpYN-2HA-OSH1-L  YEpYN-SEC22-L  YEpYC-SEC22-U  YEpYN-SCS2-L  YCpYC-SEC9(421-651)-U  YCpYC-SEC9(1-420)-U  SEC9(421-651)pEG202  YCp-H  YCpYC-SCS2-H  YCpOSH3-H  YEpYC-sec9-4-U  pYM-hphNTI  YCpGFP-U  YCpYC-SEC22-H  YCpGFP-SCS2-U | | *2µ*  *2µ*  *2µ*  *2µ*  *2µ*  *2µ*  *2µ*  *2µ*  *2µ*  *2µ*  *2µ*  *2µ*  *2µ*  *2µ*  *2µ*  *2µ*  *2µ*  *2µ*  *2µ*  *2µ*  *2µ*  *2µ*  *CEN*  *CEN*  *2µ*  *CEN*  *CEN*  *CEN*  *2µ*  *-*  *CEN*  *CEN*  *CEN* | *ADH1*  *ADH1*  *ADH1*  *ADH1*  *ADH1*  *ADH1*  *ADH1*  *ADH1*  *ADH1*  *ADH1*  *ADH1*  *ADH1*  *ADH1*  *ADH1*  *ADH1*  *ADH1*  *ADH1*  *ADH1*  *ADH1*  *ADH1*  *ADH1*  *ADH1*  *MET25*  *MET25*  *ADH1*  *ADH1*  *ADH1*  *ADH1*  *ADH1*  *-*  *MET25*  *ADH1*  *MET25* | | *-*  *-*  *SCS2*  *OSH1*  *OSH2*  *OSH3*  *OSH4*  *OSH5*  *OSH6*  *OSH7*  *OSH3(mFFAT)*  *SSO1*  *SEC9*  *SEC9*  *-*  *OSH2*  *OSH3*  *SCS2*  *OSH1*  *SEC22*  *SEC22*  *SCS2*  *SEC9(421-651)*  *SEC9(1-420)*  *Sec9(421-651)*  *-*  *SCS2*  *OSH3*  *sec9-4*  *-*  *-*  *SEC22*  *SCS2* | | | *LEU2*  *URA3*  *URA3*  *LEU2*  *LEU2*  *LEU2*  *LEU2*  *LEU2*  *LEU2*  *LEU2*  *LEU2*  *LEU2*  *LEU2*  *URA3*  *LEU2*  *LEU*  *LEU2*  *URA3*  *LEU*  *LEU2*  *URA3*  *LEU2*  *URA3*  *URA3*  *LEU2*  *HIS3*  *HIS3*  *HIS3*  *URA3*  *-*  *URA3*  *HIS*  *URA3* | | [1]  [1]  [2]  [2]  [2]  [2]  This study  This study  This study  This study  [2]  [3]  [4]  [4]  This study  This study  This study  This study  This study  This study  This study  This study  [4]  [4]  J. Jäntti  [5]  This study  This study  This study  [6]  [7]  This study  This study | |
| **Mammalian Plasmids** | | | | | | | | | | | | |
| Plasmid | Name | Species | | | Insert | | | Tag | | Source | | |
| 1140  1161  1223  1226  1227  1228  1229  1230  1231  1232  1233  1234  1235  1236  1245  1239  1269  1271  1274  1275  1276  1277  1560 | pmCherry-C1  mCherry-ORP10  VC-VAPA  VN-ORP1L  VN-ORP2  VN-ORP3  VN-ORP4L  VN-ORP5  VN-ORP6  VN-ORP7  VN-ORP8  VN-ORP9L  VN-ORP10  VN-ORP11  VN-OSBP  VC-mSNAP-25b  mCherry-ORP1L  mCherry-ORP2  mCherry-OSBP  mCherry-ORP3  mCherry-ORP4L  mCherry-ORP9L  VN-VAPA | -  human  human  human  human  human  human  human  human  human  human  human  human  human  human  mouse  human  human  human  human  human  human  human | | | -  hORP10  hVAPA  hORP1L  hORP2  hORP3  hORP4L  hORP5  hORP6  hORP7  hORP8  hORP9L  hORP10  hORP11  hOSBP  mSNAP-25b  hORP1L  hORP2  hOSBP  hORP3  hORP4L  hORP9L  hVAPA | | | mCherry  mCherry  Venus(C)  Venus(N )  Venus(N)  Venus(N)  Venus(N)  Venus(N)  Venus(N)  Venus(N)  Venus(N)  Venus(N)  Venus(N)  Venus(N)  Venus(N)  Venus(C)  mCherry  mCherry  mCherry  mCherry  mCherry  mCherry  Venus(N) | | M. Vartiainen  V. Olkkonen  [2]  [8]  [2]  [9]  [2]  [2]  [2]  [2]  [2]  [2]  [8]  [8]  [2]  [4]  This study  This study  This study  This study  This study  This study  This study | | |
| **Lentiviral Constructs** | | | | | | | | | | | | |
| Plasmid | Name | | | | Species | | Insert | | | Tag | | Source |
| BL150  BL181  BL360  BL940  BL1209  BL1210  BL1310  BL1332  BL1356  BL1466 | f(syn)w-iCreRFP-P2A  f(syn)NLS-RFP-P2Aw  f(U6)sNLS-RFPw-Scr.shClathrin  f(syn)NLS-RFP-P2A-mSNAP25w  f(syn)NLS-GFP-P2A-hORP1L-w  f(syn)NLS-GFP-P2A-hORP2-w  f(syn)-NLS-RFP-P2A-VC-hVAPA-w  f(U6)snrw-ORP2 shRNA-1  f(syn)NLS-RFP-P2A-VN-hVAPA -w  f(syn)NLS-RFP-P2A-FLAG-GSG-10kDa(VN)-hVAP-A-w | | | | mouse  human  human  human  mouse  human  human | | sc shRNA  SNAP-25  ORP1L  ORP2  VAPA  ORP2 shRNA  VAPA  VAPA | | | Venus(C)  Venus(N)  FLAG-Venus(N) | | [10]  [10]  [11]  This study  This study  This study  This study  This study  This study  This study |
| **Bacteria Plasmids** | | | | | | | | | | | | |
| Plasmid | Name | | Insert | | | Marker | | | | Source | | |
| 977  1300  1304  1338  1457  1509  1561  1564  B3760 | pGEX  pFOLD-ORP2  pFOLD  VAPA(ΔTM)pGEX4T1  VAPA(ΔTM)pHAT5  pHAT3  SEC9(421-651)pGEX  Osh3pHAT3  GST-SNAP25 | | -  *hORP2*  *-*  *VAPA(ΔTM)*  *VAPA(ΔTM)*  *SEC9(421-651)*  *OSH3*  *mSNAP-25* | | | Amp  Amp  Amp  Amp  Amp  Amp  Amp  Amp  Amp | | | | V. Olkkonen  ORP-VAP  J. Peränen  V. Olkkonen  V. Olkkonen  J. Peränen  This study  This study  [4] | | |

**Supplementary Table S3. Statistical Analysis**

| Figure # | Panel | Parameter measured | Statistical Test | Number of replicates (N) | Sample name | Cells per replicate | Total # cells (n) | Mean± SEM | Comparison | | p-value |  |
| --- | --- | --- | --- | --- | --- | --- | --- | --- | --- | --- | --- | --- |
| Figure 1 | 1C | YNOsh1p-YcScs2p BiFC signal intenity | Kruskal-Wallis test followed by Dunn's multiple comparison test | 2 | wt | 15+16+15+24+15+16 | 101 | 1.00+/-0.05 | wt vs. |  |  |  |
|  |  |  |  |  | *Δsso1* | 18+16 | 34 | 1.02+/-0.08 |  | *Δsso1* | >0.9999 |  |
|  |  |  |  |  | *Δsso2* | 16+12 | 28 | 1.03+/-0.09 |  | *Δsso2* | >0.9999 |  |
|  |  |  |  |  | *sso2-1 Δsso1* | 15+16 | 31 | 0.99+/-0.09 |  | *sso2-1 Δsso1* | >0.9999 |  |
|  |  |  |  |  | *sec9-4* | 15+16 | 31 | 0.14+/-0.01 |  | *sec9-4* | <0.0001 | *** |
|  |  |  |  |  | *Δscn1* | 15+24 | 39 | 0.33+/-0.03 |  | *Δscn1* | <0.0001 | *** |
|  | 1D | YNOsh2p-YcScs2p BiFC signal intenity | Kruskal-Wallis test followed by Dunn's multiple comparison test | 2 | wt | 19+20+18+21+12+16 | 106 | 1.00+/-0.03 | wt vs. |  |  |  |
|  |  |  |  |  | *Δsso1* | 22+14 | 36 | 1.10+/-0.07 |  | *Δsso1* | >0.9999 |  |
|  |  |  |  |  | *Δsso2* | 13+18 | 31 | 1.12+/-0.07 |  | *Δsso2* | >0.9999 |  |
|  |  |  |  |  | *sso2-1 Δsso1* | 18+21 | 39 | 0.35+/-0.03 |  | *sso2-1 Δsso1* | <0.0001 | *** |
|  |  |  |  |  | *sec9-4* | 18+21 | 39 | 0.04+/-0.02 |  | *sec9-4* | <0.0001 | *** |
|  |  |  |  |  | *Δscn1* | 19+20 | 39 | 1.11+/-0.07 |  | *Δscn1* | >0.9999 |  |
|  | 1E | YNOsh3p-YcScs2p BiFC signal intenity | Kruskal-Wallis test followed by Dunn's multiple comparison test | 2 | wt | 17+22+13+21+10+16 | 99 | 1.00+/-0.04 | wt vs. |  |  |  |
|  |  |  |  |  | *Δsso1* | 14+15 | 29 | 1.01+/-0.09 |  | *Δsso1* | >0.9999 |  |
|  |  |  |  |  | *Δsso2* | 13+18 | 31 | 1.03+/-0.07 |  | *Δsso2* | >0.9999 |  |
|  |  |  |  |  | *sso2-1 Δsso1* | 17+22 | 39 | 0.14+/-0.01 |  | *sso2-1 Δsso1* | <0.0001 | *** |
|  |  |  |  |  | *sec9-4* | 11+16 | 27 | 0.03+/-0.01 |  | *sec9-4* | <0.0001 | *** |
|  |  |  |  |  | *Δscn1* | 17+22 | 39 | 1.00+/-0.04 |  | *Δscn1* | >0.9999 |  |
| Figure 5 | 5C | BiFC signal intenity | Mann-Whitney test | 2 | YCScs2-YNScs2 vector | 23+27 | 50 | 1.00+/-0.07 | vector vs. |  |  |  |
|  |  |  |  |  | YCScs2-YNScs2 *OSH3* | 24+38 | 62 | 1.46+/-0.13 |  | *OSH3* | 0.0418 | * |
|  |  |  |  |  | YCScs2-YNSec9 vector | 24+25 | 49 | 1.00+/-0.04 |  |  |  |  |
|  |  |  |  |  | YCScs2-YNSec9 *OSH3* | 27+26 | 53 | 1.37+/-0.08 |  | *OSH3* | 0.0009 | *** |
|  |  |  |  |  | YCSec22-YNSso1 vector | 28+22 | 50 | 1.00+/-0.08 |  |  |  |  |
|  |  |  |  |  | YCSec22-YNSso1 *OSH3* | 28+22 | 50 | 1.65+/-0.15 |  | *OSH3* | 0.001 | ** |
| Figure 6 | 6B | VAPA-VAPA patches/cell | Kruskal-Wallis test followed by Dunn's multiple comparison test | 2-3 | sc DIV7 | 20+10 | 30 | 14.27+/-1.36 | sc vs. |  |  |  |
|  |  |  |  |  | ORP2 KD DIV7 | 20+10 | 30 | 8.4+/-1.27 |  | ORP2 KD | 0.0093 | ** |
|  |  |  |  |  | sc DIV14 | 3+14+11 | 28 | 15.46+/-2.06 |  |  |  |  |
|  |  |  |  |  | ORP2 KD DIV14 | 6+15+9 | 30 | 6.6+/-0.84 |  | ORP2 KD | 0.0007 | *** |
|  | 6E | norm FLAG-VAPA co-IP | N too small | 3 | sc VAPA |  |  | 1.00+/-0.00 |  |  |  |  |
|  |  |  |  |  | ORP2 KD VAPA |  |  | 0.55+/-0.14 |  |  |  |  |
|  |  |  |  |  | sc Stx1A |  |  | 1.00+/-0.00 |  |  |  |  |
|  |  |  |  |  | ORP2 KD Stx1A |  |  | 0.27+/-0.05 |  |  |  |  |
|  | 6G | norm FLAG-VAPA co-IP | N too small | 3 | sc VAPA |  |  | 1.00+/-0.00 |  |  |  |  |
|  |  |  |  |  | ORP2 KD VAPA |  |  | 0.96+/-0.07 |  |  |  |  |
|  |  |  |  |  | sc Stx1A |  |  | 1.00+/-0.00 |  |  |  |  |
|  |  |  |  |  | ORP2 KD Stx1A |  |  | 0.66+/-0.22 |  |  |  |  |
| Figure 7 | 7C | dendritic lenght (µm) | unpaired t-test | 3 | sc | 15+10+10 | 35 | 1426+/-81.21 | sc vs. |  |  |  |
|  |  |  |  |  | ORP2 KD | 15+10+10 | 35 | 796+/-53.84 |  | ORP2 KD | <0.0001 | *** |
|  | 7D | vGlut dots (nr/cell) | Mann-Whitney test | 3 | sc | 15+10+10 | 35 | 160.6+/-19.07 | sc vs. |  |  |  |
|  |  |  |  |  | ORP2 KD | 14+9+8 | 31 | 79+/-8.38 |  | ORP2 KD | 0.0010 | *** |
|  | 7E | vGlut1 dot size (µm2) | unpaired t-test | 3 | sc | 15+10+10 | 35 | 0.87+/-0.07 | sc vs. |  |  |  |
|  |  |  |  |  | ORP2 KD | 14+9+8 | 31 | 1.02+/-0.09 |  | ORP2 KD | 0.1589 |  |
| Figure 8 | 8B | Hsp150 secretion efficiency | N to small | 3 | wt + v |  |  | 1.00+/-0.00 |  |  |  |  |
|  |  |  |  |  | wt + *OSH3* |  |  | 0.34+/-0.20 |  |  |  |  |
|  |  |  |  |  | wt |  |  | 1.00+/-0.00 |  |  |  |  |
|  |  |  |  |  | *Δosh1-3* |  |  | 1.60+/-0.10 |  |  |  |  |
|  | 8C | Hsp150 secretion efficiency | N too small | 3 | *sec1-1* + v |  |  | 1.00+/-0.00 |  |  |  |  |
|  |  |  |  |  | *sec1-1* + *OSH3* |  |  | 0.30+/-0.20 |  |  |  |  |
|  |  |  |  |  | *sec9-4* + v |  |  | 1.00+/-0.00 |  |  |  |  |
|  |  |  |  |  | *sec9-4* + *OSH3* |  |  | 1,28+/-0.25 |  |  |  |  |
|  |  |  |  |  | *sec9-4 Δpep4* + v |  |  | 1.00+/-0.00 |  |  |  |  |
|  |  |  |  |  | *sec9-4 Δpep4* + *OSH3* |  |  | 0.29+/-0.15 |  |  |  |  |
|  | 8C’ | Hsp150 secretion efficiency | N too small | 3 | *sec9-4* + v |  |  | 1.00+/-0.00 |  |  |  |  |
|  |  |  |  |  | *sec9-4 Δpep4* + v |  |  | 0.48+/-0.11 |  |  |  |  |
|  | 8D | Hsp150 secretion efficiency | N too small | 3 | *Δosh1-3* + v |  |  | 1.00+/-0.00 |  |  |  |  |
|  |  |  |  |  | *Δosh1-3* + *OSH3(wt)* |  |  | 0.59+/-0.06 |  |  |  |  |
|  |  |  |  |  | *Δosh1-3* + *OSH3 (mFFAT)* |  |  | 0.93+/-0,12 |  |  |  |  |
| Figure S1 | S1A | YNOsh1p-YcScs2p BiFC signal intenity | Kruskal-Wallis test followed by Dunn's multiple comparison test | 2 | wt | 16+23 | 39 | 1.00+/-0.07 | wt vs. |  |  |  |
|  |  |  |  |  | *sec4-8* | 18+21 | 39 | 1.09+/-0.07 |  | *sec4-8* | >0.9999 |  |
|  |  |  |  |  | *sec18-1* | 16+23 | 39 | 1.06+/-0.07 |  | *sec18-1* | >0.9999 |  |
|  |  |  |  |  | *Δmso1* | 13+21 | 34 | 1.04+/-0.09 |  | *Δmso1* | >0.9999 |  |
|  |  |  |  |  | *sec1-1* | 13+21 | 34 | 1.03+/-0.08 |  | *sec1-1* | >0.9999 |  |
|  |  |  |  |  | *Δsro7* | 18+21 | 39 | 1.09+/-0.08 |  | *Δsro7* | >0.9999 |  |
|  | S1B | YNOsh2p-YcScs2p BiFC signal intenity | Kruskal-Wallis test followed by Dunn's multiple comparison test | 2 | wt | 19+20 | 39 | 1.00+/-0.05 | wt vs. |  |  |  |
|  |  |  |  |  | *sec4-8* | 11+17 | 28 | 1.01+/-0.07 |  | *sec4-8* | >0.9999 |  |
|  |  |  |  |  | *sec18-1* | 19+20 | 39 | 0.99+/-0.06 |  | *sec18-1* | >0.9999 |  |
|  |  |  |  |  | *Δmso1* | 19+20 | 39 | 1.04+/-0.08 |  | *Δmso1* | >0.9999 |  |
|  |  |  |  |  | *sec1-1* | 19+20 | 39 | 1.07+/-0.06 |  | *sec1-1* | >0.9999 |  |
|  |  |  |  |  | *Δsro7* | 19+20 | 39 | 1.03+/-0.07 |  | *Δsro7* | >0.9999 |  |
|  | S1C | YNOsh3p-YcScs2p BiFC signal intenity | Kruskal-Wallis test followed by Dunn's multiple comparison test | 2 | wt | 17+22 | 39 | 1.00+/-0.10 | wt vs. |  |  |  |
|  |  |  |  |  | *sec4-8* | 17+22 | 39 | 1.04+/-0.08 |  | *sec4-8* | >0.9999 |  |
|  |  |  |  |  | *sec18-1* | 17+22 | 39 | 0.93+/-0.08 |  | *sec18-1* | >0.9999 |  |
|  |  |  |  |  | *Δmso1* | 15+17 | 32 | 1.01+/-0.08 |  | *Δmso1* | >0.9999 |  |
|  |  |  |  |  | *sec1-1* | 17+22 | 39 | 1.18+/-0.06 |  | *sec1-1* | 0.0984 |  |
|  |  |  |  |  | *Δsro7* | 17+22 | 39 | 1.07+/-0.07 |  | *Δsro7* | >0.9999 |  |
| Figure S2 | S2b | norm co-IP with HA-Osh3p (Sec9p upper row, Sso1/2p lower row) | N too small | 3 | wt |  |  | 1.00+/-0.00 |  |  |  |  |
|  |  |  |  |  |  |  |  | 1.00+/-0.00 |  |  |  |  |
|  |  |  |  |  | *sec9-4* |  |  | 0.00+/-0.00 |  |  |  |  |
|  |  |  |  |  |  |  |  | 0.00+/-0.00 |  |  |  |  |
|  |  |  |  |  | *sso2-1 Δsso1* |  |  | 0.32+/-0.15 |  |  |  |  |
|  |  |  |  |  |  |  |  | 0.50+/-0.07 |  |  |  |  |
|  |  |  |  |  | *Δsnc1* |  |  | 0.95+/-0.05 |  |  |  |  |
|  |  |  |  |  |  |  |  | 0.91+/-0.07 |  |  |  |  |
|  |  |  |  |  | *sec4-8* |  |  | 1.69+/-0.21 |  |  |  |  |
|  |  |  |  |  |  |  |  | 1.21+/-0.18 |  |  |  |  |
| Figure S3 | S3B | norm ORP2/β-Tubulin | N too small | 3 | sc |  |  | 1.00+/-0.00 |  |  |  |  |
|  |  |  |  |  | ORP2 KD |  |  | 0.39+/-0.11 |  |  |  |  |
|  | S3D | norm ORP2/β-Tubulin | N too small | 3 | sc |  |  | 1.00+/-0.00 |  |  |  |  |
|  |  |  |  |  | ORP2 KD |  |  | 0.17+/-0.04 |  |  |  |  |
| Figure S5 | S5B | dendritic lenght (µm) | Mann-Whitney test | 3 | sc |  | 28 | 1245+/-108.1 | sc vs. |  |  |  |
|  |  |  |  |  | ORP2 KD |  | 25 | 1097+/-85.53 |  | ORP2 KD | 0.4415 |  |
|  | S5C | vGlut dots (nr/cell) | Mann-Whitney test | 3 | sc |  | 30 | 193.2+/-22.24 | sc vs. |  |  |  |
|  |  |  |  |  | ORP2 KD |  | 27 | 219.3+/-24 |  | ORP2 KD | 0.4021 |  |
|  | S5D | vGlut1 dot zize (µm2) | unpaired t-test | 3 | sc |  | 30 | 1.29+/-0,08 | sc vs. |  |  |  |
|  |  |  |  |  | ORP2 KD |  | 27 | 1.25+/-0.08 |  | ORP2 KD | 0.7233 |  |

**References**

1. Weber-Boyvat M, Li S, Skarp KP, Olkkonen VM, Yan D, Jantti J (2015) Bimolecular fluorescence complementation (BiFC) technique in yeast Saccharomyces cerevisiae and mammalian cells. Methods Mol Biol 1270:277-288. doi:10.1007/978-1-4939-2309-0_20

2. Weber-Boyvat M, Kentala H, Peranen J, Olkkonen VM (2015) Ligand-dependent localization and function of ORP-VAP complexes at membrane contact sites. Cell Mol Life Sci 72 (10):1967-1987. doi:10.1007/s00018-014-1786-x

3. Weber M, Chernov K, Turakainen H, Wohlfahrt G, Pajunen M, Savilahti H, Jantti J (2010) Mso1p regulates membrane fusion through interactions with the putative N-peptide-binding area in Sec1p domain 1. Mol Biol Cell 21 (8):1362-1374. doi:10.1091/mbc.E09-07-0546

4. Weber-Boyvat M, Chernov KG, Aro N, Wohlfahrt G, Olkkonen VM, Jantti J (2016) The Sec1/Munc18 Protein Groove Plays a Conserved Role in Interaction with Sec9p/SNAP-25. Traffic 17 (2):131-153. doi:10.1111/tra.12349

5. Mumberg D, Muller R, Funk M (1994) Regulatable promoters of Saccharomyces cerevisiae: comparison of transcriptional activity and their use for heterologous expression. Nucleic Acids Res 22 (25):5767-5768. doi:10.1093/nar/22.25.5767

6. Janke C, Magiera MM, Rathfelder N, Taxis C, Reber S, Maekawa H, Moreno-Borchart A, Doenges G, Schwob E, Schiebel E, Knop M (2004) A versatile toolbox for PCR-based tagging of yeast genes: new fluorescent proteins, more markers and promoter substitution cassettes. Yeast 21 (11):947-962. doi:10.1002/yea.1142

7. Niedenthal RK, Riles L, Johnston M, Hegemann JH (1996) Green fluorescent protein as a marker for gene expression and subcellular localization in budding yeast. Yeast 12 (8):773-786. doi:Doi 10.1002/(Sici)1097-0061(19960630)12:8<773::Aid-Yea972>3.3.Co;2-C

8. Nissila E, Ohsaki Y, Weber-Boyvat M, Perttila J, Ikonen E, Olkkonen VM (2012) ORP10, a cholesterol binding protein associated with microtubules, regulates apolipoprotein B-100 secretion. Biochim Biophys Acta 1821 (12):1472-1484. doi:10.1016/j.bbalip.2012.08.004

9. Weber-Boyvat M, Kentala H, Lilja J, Vihervaara T, Hanninen R, Zhou Y, Peranen J, Nyman TA, Ivaska J, Olkkonen VM (2015) OSBP-related protein 3 (ORP3) coupling with VAMP-associated protein A regulates R-Ras activity. Exp Cell Res 331 (2):278-291. doi:10.1016/j.yexcr.2014.10.019

10. Vardar G, Chang S, Arancillo M, Wu YJ, Trimbuch T, Rosenmund C (2016) Distinct Functions of Syntaxin-1 in Neuronal Maintenance, Synaptic Vesicle Docking, and Fusion in Mouse Neurons. J Neurosci 36 (30):7911-7924. doi:10.1523/JNEUROSCI.1314-16.2016

11. Kononenko NL, Puchkov D, Classen GA, Walter AM, Pechstein A, Sawade L, Kaempf N, Trimbuch T, Lorenz D, Rosenmund C, Maritzen T, Haucke V (2014) Clathrin/AP-2 mediate synaptic vesicle reformation from endosome-like vacuoles but are not essential for membrane retrieval at central synapses. Neuron 82 (5):981-988. doi:10.1016/j.neuron.2014.05.007
