# Supplementary figures and images for: ORP/Osh mediate cross-talk between ER-plasma membrane contact site components and plasma membrane SNAREs
Source: Cell Mol Life Sci. 2020 Jul 30;78(4):1689–708. doi: 10.1007/s00018-020-03604-w (PMC7904734; doi:10.1007/s00018-020-03604-w)

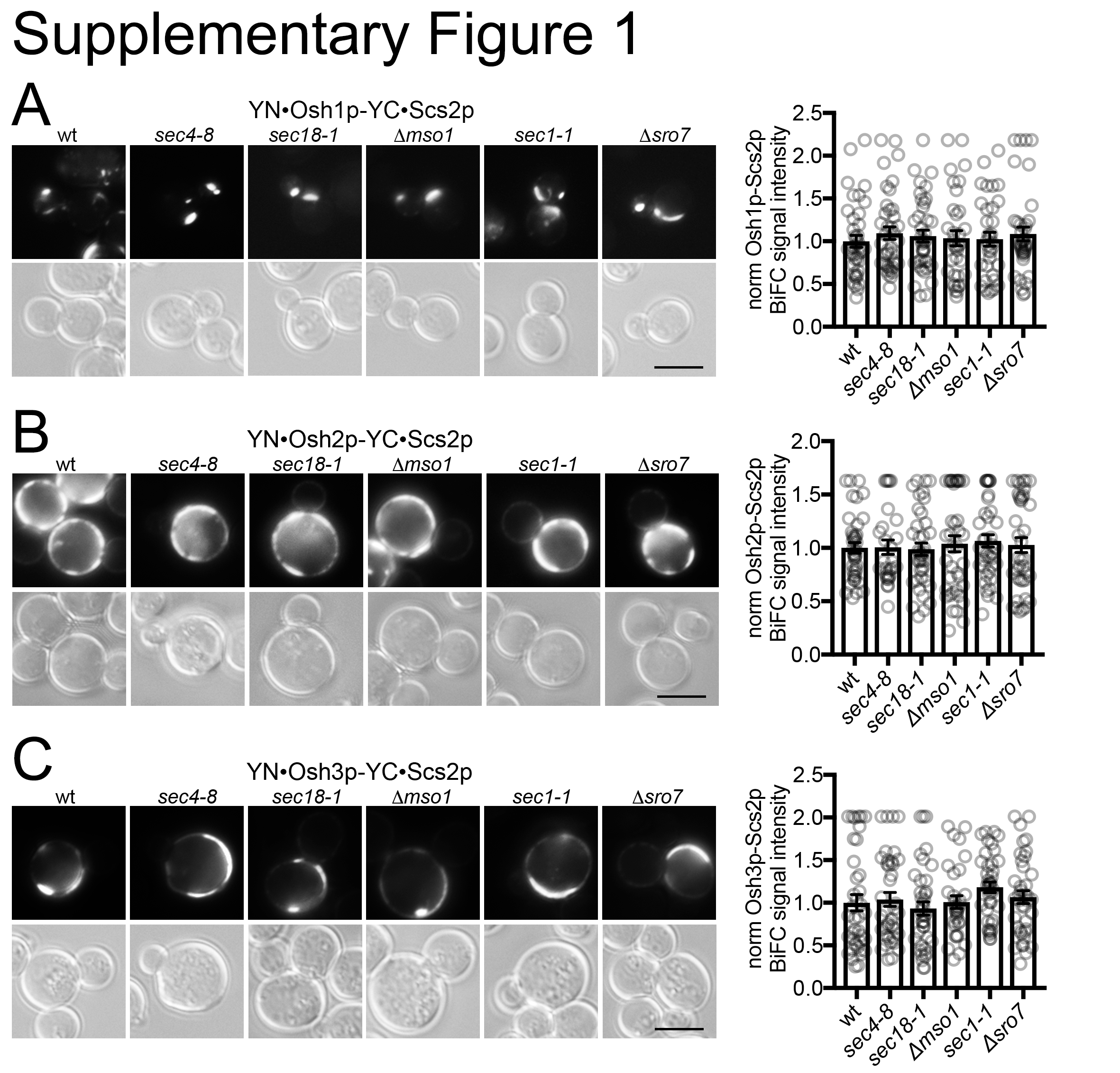

Supplement: Supplementary file 2 — Supplementary file2 Supplementary Figure 1. Mutants defective in exocytic SNARE complex assembly or disassembly do not affect Oshp-Scs2p BiFC interaction. Live cell imaging of indicated vegetatively grown haploid cells (wt, Y1; sec4-8, Y19; sec18-1, Y13; Δmso1, Y14; sec1-1, Y15; Δsro7, Y17) expressing YFP(N)·Osh1p (1362, A), YFP(N)·Osh2p (1363, B) or YFP(N)·Osh3p (1364, C) in combination with YFP(C)·Scs2p (1360). BiFC interactions were monitored by fluorescence microscopy in in a minimum of 28 cells grown at 24°C. The data represent mean ± SEM. Scale bar, 5 µm(TIF 15707 kb). [file 18_2020_3604_MOESM2_ESM.tif]

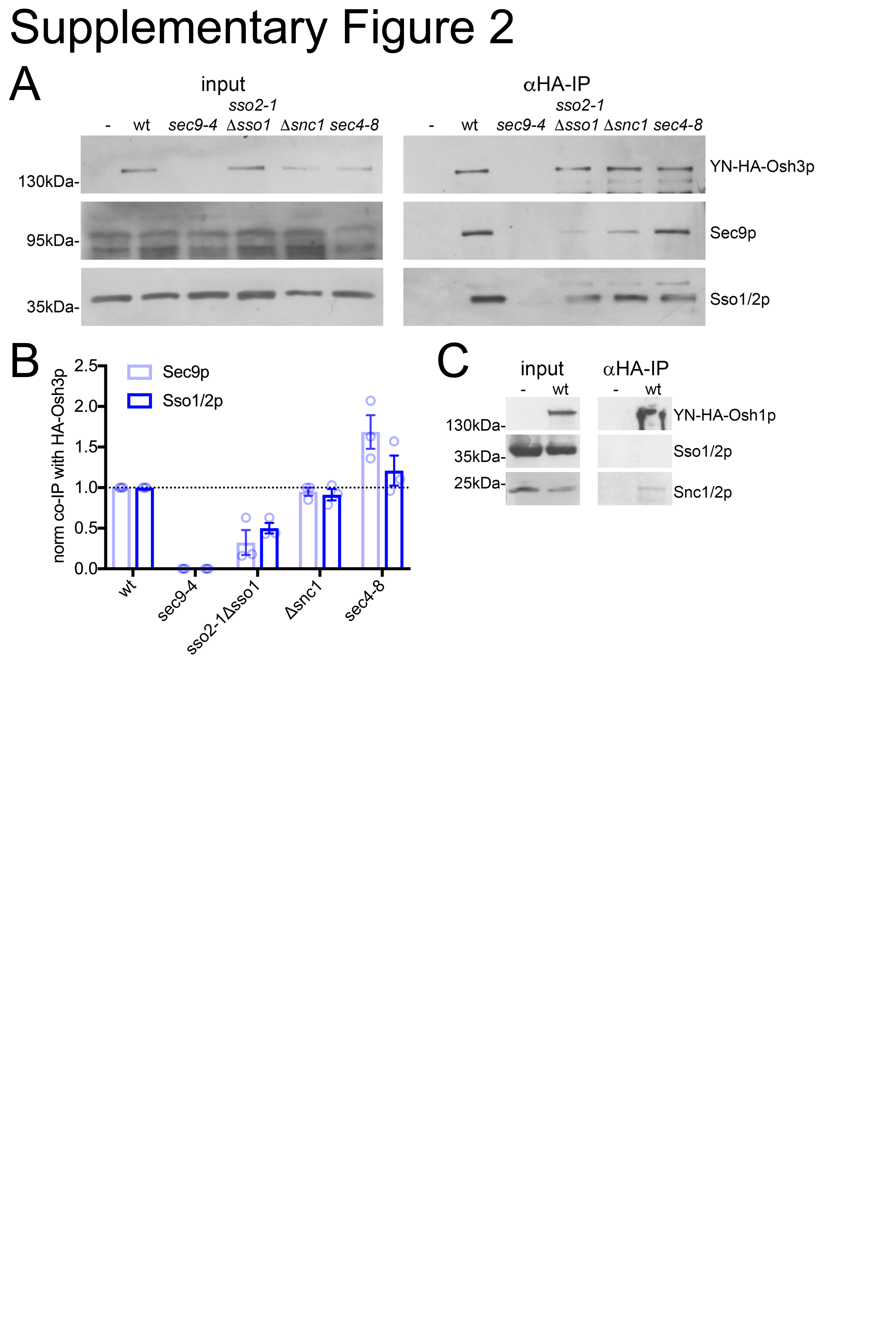

Supplement: Supplementary file 3 — Supplementary file3 Supplementary Figure 2. The plasma membrane SNAREs Sec9p and Sso1/2p co-immunoprecipitate with Osh3p, while Osh1p associates with Snc1/2p. (A). Indicated yeast cells (wt, Y1; sec9-4, Y6; sso2-1 Δsso1, Y7; Δscn1, Y12; sec4-8, Y19) expressing either empty vector (-; 1356) or YFP(N)·HA·Osh3p (1477) were grown until OD600 = 1, lysed, and subjected to anti-HA immunoprecipitations. Immunoprecipitates and lysates were analyzed by Western blotting with anti-HA, -Sec9p, and -Sso1/2p antibodies. (B) Quantification of three independent experiments of immunoprecipitations shown in (A). The data represent mean ± SEM. (C) Wt yeast cells (Y1) expressing either empty vector (-; 1356) or YFP(N)·HA·Osh1p (1513) were grown until OD600 = 1, lysed, and subjected to anti-HA immunoprecipitations. Immunoprecipitates and lysates were analyzed by Western blotting with anti-HA, -Sso1/2p, and -Snc1p antibodies(TIF 24549 kb). [file 18_2020_3604_MOESM3_ESM.tif]

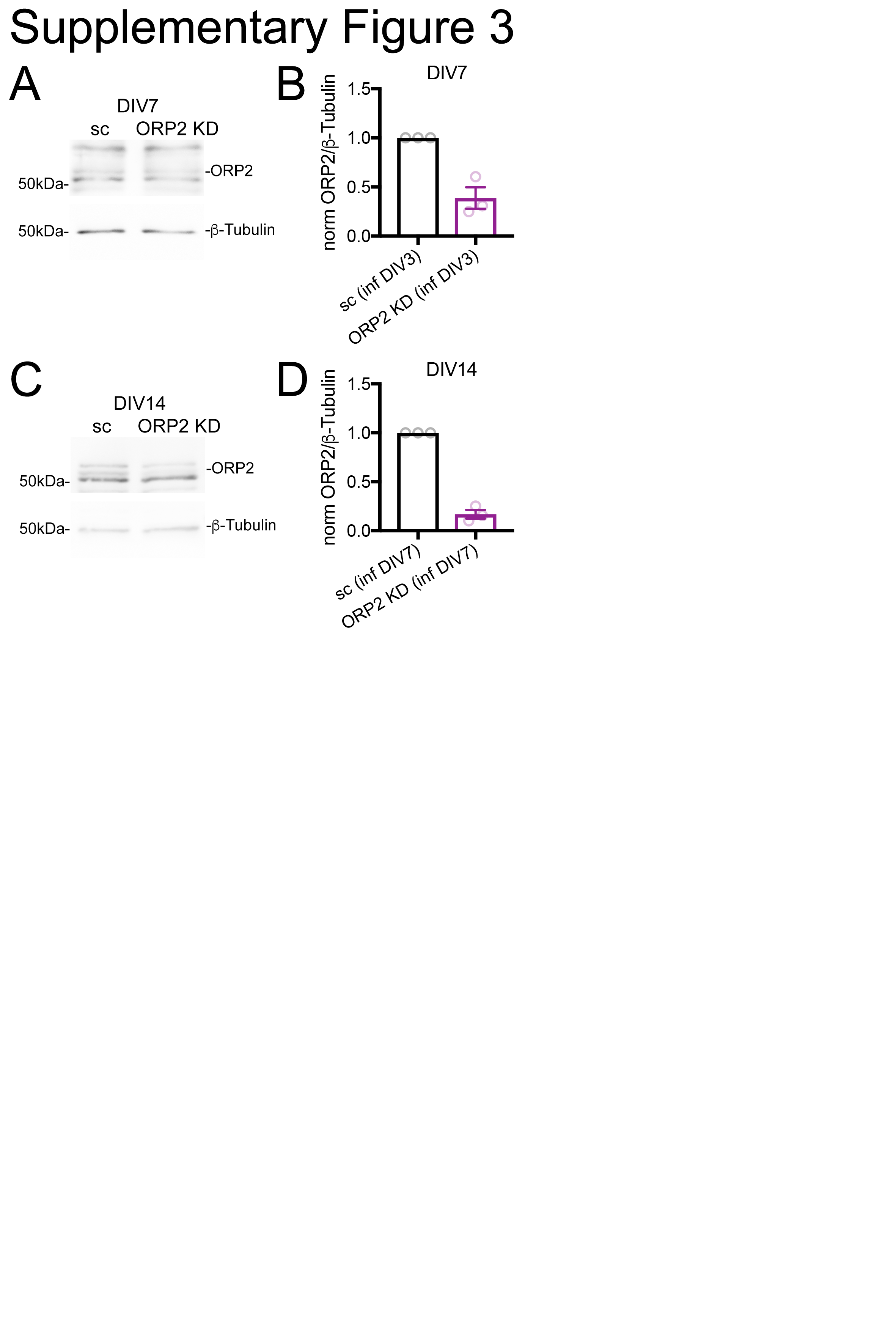

Supplement: Supplementary file 4 — Supplementary file4 Supplementary Figure 3. ORP2 Knock-down efficiency in mice hippocampal neurons. (A) SDS lysates of lentivirus-based ORP2 KD in mice hippocampal neurons at DIV 7. Cells were transduced with scrambled shRNA (sc, BL360) or shRNA against ORP2 (ORP2 KD, BL1332) at DIV 3 and lysed at DIV 7. SDS lysates were analyzed by Western blotting and detection and anti-ORP2 and -β-Tubulin antibodies. (B) Quantification of ORP2 KD efficiency in three independed cultures as in (A). (C) SDS lysates of lentivirus-based ORP2 KD in mice hippocampal neurons at DIV 14. Cells were transduced with scrambled shRNA (sc, BL360) or shRNA against ORP2 (ORP2 KD, BL1332) at DIV 7 and lysed at DIV 14. SDS lysates were analyzed by Western blotting and detection and anti-ORP2 and -β-Tubulin antibodies. (D) Quantification of ORP2 KD efficiency in three independed cultures as in (C)(TIF 24548 kb). [file 18_2020_3604_MOESM4_ESM.tif]

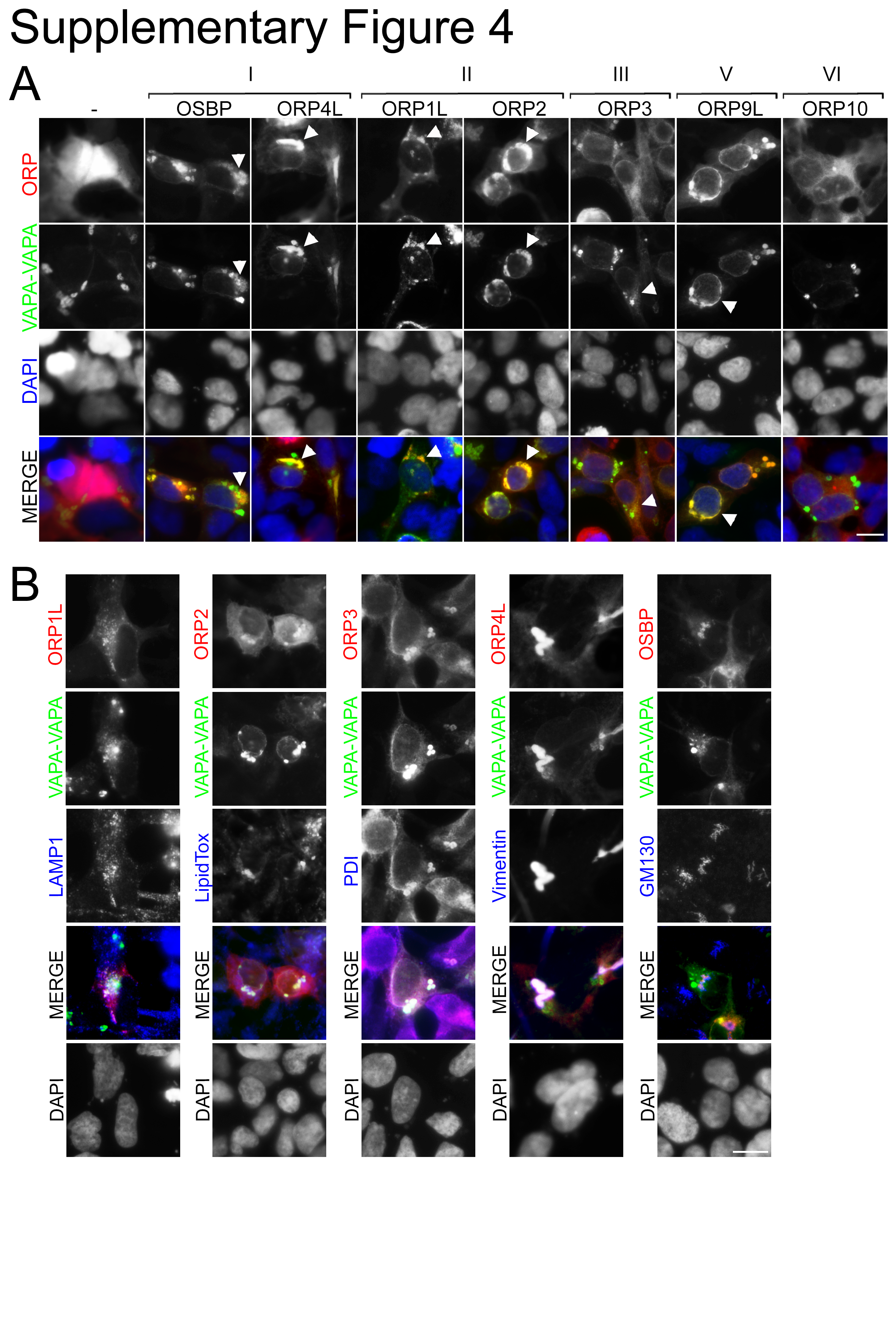

Supplement: Supplementary file 5 — Supplementary file5 Supplementary Figure 4. Overexpression of certain ORPs causes VAPA oligomer re-localization in vivo. (A) Fluorescence imaging of HEK293 cells transfected with plasmids expressing Venus(N)·VAPA (1560) and Venus(C)·VAPA (1223) in combination with indicated mCherry·ORP constructs (-, 1140; OSBP, 1274; ORP4L, 1276; ORP1L, 1269; ORP2, 1271; ORP3, 1275; ORP9L, 1277; ORP10, 1161). Arrowheads point to VAPA oligomers localizing to the Golgi complex (OSBP), Vimentin intermediate filament (ORP4L), late endosomes (ORP1L) and lipid droplets (ORP2). Scale bar 10µm. (B) Fluorescence imaging as in (A) with additional staining with anti-PDI antibody for ER, anti-LAMP1 antibody for late endosomes, LipidTox for lipid droplets, anti-Vimentin antibody for Vimentin intermediate filament and anti-GM130 antibody for Golgi complex. Scale bar, 10 µm(TIF 24553 kb). [file 18_2020_3604_MOESM5_ESM.tif]

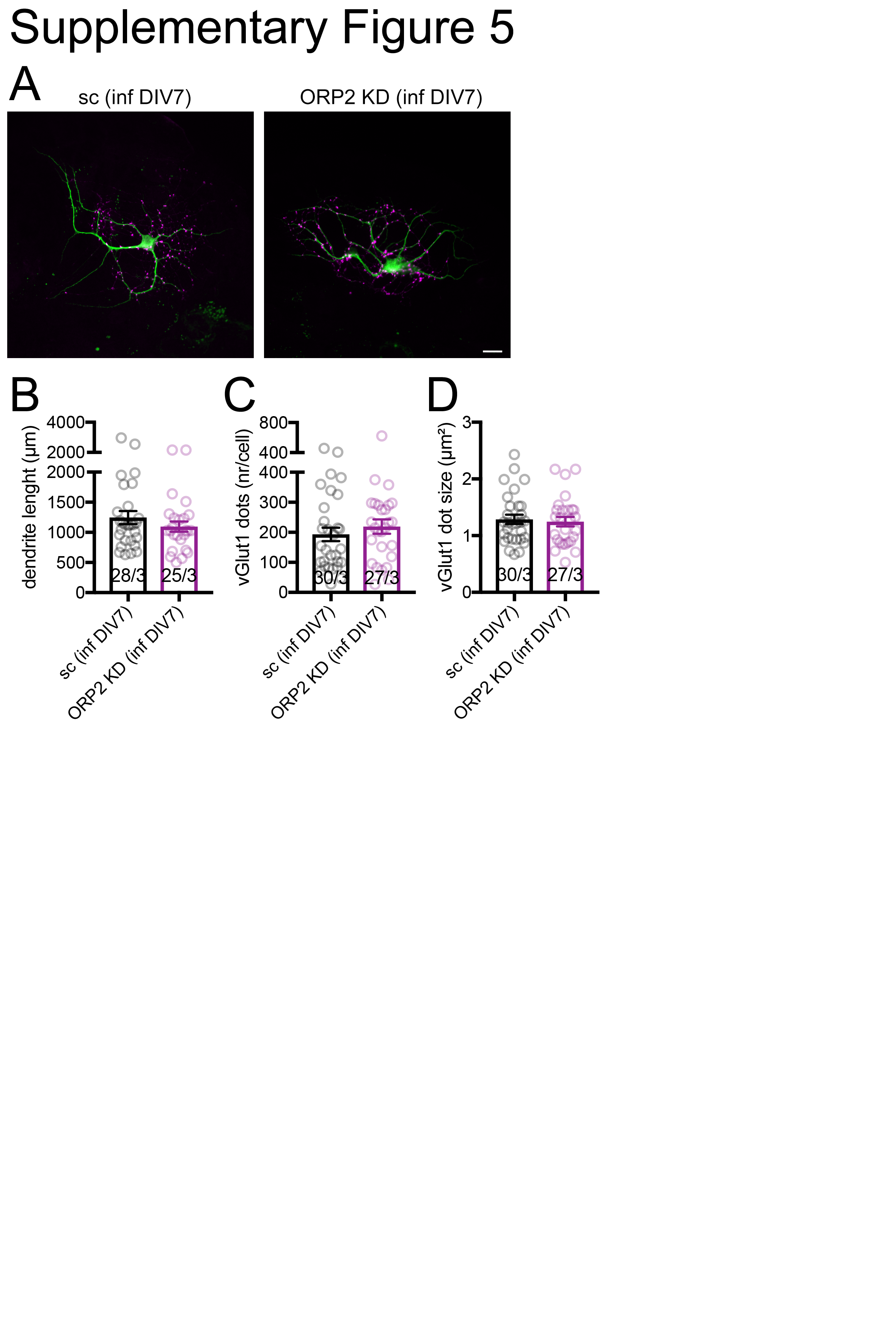

Supplement: Supplementary file 6 — Supplementary file6 Supplementary Figure 5. ORP2 knock-down at DIV 7 does not alter neuronal cell morphology. (A) Immunofluorescence imaging of wt mice hippocampal neurons with lentivirus-based knock-down of ORP2 at DIV 7 (sc BL360, ORP2 KD BL1332). Cells were fixed at DIV 14 and immunofluorescence staining against vGlut1 (magenta) and MAP2 (green) performed. Scale bar 40µm. (B) Quantification of dendrite length per cell (stained with MAP2) in (A). (C and D) Quantification of vGlut1 dot number and size per cell in (A). Shown is mean +/-SEM, number of cells/independent cultures(TIF 24549 kb). [file 18_2020_3604_MOESM6_ESM.tif]
